# Intermediate-term emotional bookkeeping is necessary for long-term reciprocal grooming partner preferences in an agent-based model of macaque groups

**DOI:** 10.7717/peerj.1488

**Published:** 2016-01-05

**Authors:** Ellen Evers, Han de Vries, Berry M. Spruijt, Elisabeth H.M. Sterck

**Affiliations:** 1 Department of Animal Ecology, Utrecht University, Utrecht, The Netherlands; 2 Animal Science Department, Biomedical Primate Research Center, Rijswijk, The Netherlands

**Keywords:** Emotional bookkeeping, Macaques, Agent-based model, Socio-emotional memory, Long-term affiliative relationships, Reciprocity, Social bonds, EMO-model

## Abstract

Whether and how primates are able to maintain long-term affiliative relationships is still under debate. Emotional bookkeeping (EB), the partner-specific accumulation of emotional responses to earlier interactions, is a candidate mechanism that does not require high cognitive abilities. EB is difficult to study in real animals, due to the complexity of primate social life. Therefore, we developed an agent-based model based on macaque behavior, the EMO-model, that implements arousal and two emotional dimensions, anxiety-FEAR and satisfaction-LIKE, which regulate social behavior. To implement EB, model individuals assign dynamic LIKE attitudes towards their group members, integrating partner-specific emotional responses to earlier received grooming episodes. Two key parameters in the model were varied to explore their effects on long-term affiliative relationships: (1) the timeframe over which earlier affiliation is accumulated into the LIKE attitudes; and (2) the degree of partner selectivity. EB over short and long timeframes gave rise to low variation in LIKE attitudes, and grooming partner preferences were only maintained over one to two months. Only EB over intermediate-term timeframes resulted in enough variation in LIKE attitudes, which, in combination with high partner selectivity, enables individuals to differentiate between regular and incidental grooming partners. These specific settings resulted in a strong feedback between differentiated LIKE attitudes and the distribution of grooming, giving rise to strongly reciprocated partner preferences that could be maintained for longer periods, occasionally up to one or two years. Moreover, at these settings the individual’s internal, socio-emotional memory of earlier affiliative episodes (LIKE attitudes) corresponded best to observable behavior (grooming partner preferences). In sum, our model suggests that intermediate-term LIKE dynamics and high partner selectivity seem most plausible for primates relying on emotional bookkeeping to maintain their social bonds.

## Introduction

Group-living animals have varied social relationships with their group members. In primates, this variation has been extensively investigated and different types of relationships are distinguished, for example dominance relationships, kinship and affiliative relationships (social bonds). Affiliative relationships among kin usually show high rates of proximity and mutually high grooming frequencies, and are therefore considered of higher quality than non-kin relationships with scarce grooming (e.g., Silk, Alberts & Altmann, 2006a; Silk, Altmann & Alberts, 2006b). However, non-kin friendly relationships can vary as well in their quality based on high or low rates of proximity and grooming (Silk, 2002; Massen, Sterck & De Vos, 2010; Massen & Sterck, 2013). The quality of social relationships may not only be affected by, but may also affect patterns of social behavior (Hinde, 1976). Moreover, social relationships provide a substrate for evolution, as strong bonds with kin and non-kin partners may provide fitness benefits (baboons (Silk, Alberts & Altmann, 2003; Silk et al., 2009; Silk et al., 2010a), feral horses (Cameron, Setsaas & Linklater, 2009), bottlenose dolphins (Frère et al., 2010) and rhesus macaques (Kulik et al., 2012; Massen et al., 2012)). Therefore, the nature and number of relationships are important features of group living animals.

Social relationships are defined as consistent patterns in social interactions between two individuals and are the elements underlying the social (group) structure (Hinde, 1976). Whether such relationships only exist in the mind of the observing researcher (Barrett & Henzi, 2002; Barrett, Henzi & Rendall, 2007) or are also represented in the mind of the interacting animals themselves (Schino & Aureli, 2009) is debated. Some interaction patterns, which may be abstracted by the observing researcher as relationships or social structure, may arise without the participants’ knowledge of such relationships or social structure. For instance, interaction patterns may simply arise as a consequence of non-random proximity patterns (De Waal & Luttrell, 1988) that emerge from the movements of the individuals relative to each other. Model studies have shown that spatial centrality of dominants, a socio-spatial group pattern often reported in primates (Evers et al., 2011), may simply arise as a result of differential fleeing, avoidance, velocity or social vigilance (Hemelrijk, 2000; Bryson, Ando & Lehmann, 2007; Evers et al., 2011; Evers et al., 2012), although the model individuals had no spatial preference for any particular location or any particular group member whatsoever. Such spatial patterning determines who encounters whom and may thus also determine the interaction structure, e.g., resulting in more frequent interactions between similar-ranking individuals compared to distant-ranking (Hemelrijk, 2000; Evers et al., 2011; Evers et al., 2012). However, in the agent based EMO-model, proximity patterns alone do not generate individual-specific reciprocal grooming relationships (Evers et al., 2015), suggesting that non-random proximity patterns are not sufficient to generate friendships.

Research suggests that primates are able to recognize their social relationships with others (Cheney & Seyfarth, 1990) and even relationships among others (e.g., kinship (Cheney & Seyfarth, 1986; Cheney & Seyfarth, 1990; Dasser, 1988); dominance (Cheney & Seyfarth, 1982; Bergman et al., 2003; Massen et al., 2011); pair bonds (Kummer, Götz & Angst, 1974)). This has led to the common view that primate social relationships are not only distinguished by researchers, but also discerned by primates. Different candidate mechanisms have been proposed to explain how individuals may internally represent their relationships with others. A cognitively relatively simple mechanism is emotional bookkeeping (Aureli & Schaffner, 2002; Aureli & Schino, 2004; Schino & Aureli, 2009): the emotional impact of previous interactions with a specific partner is integrated in an emotional valuation of the relationship with this particular partner, i.e., an emotional attitude, and may affect subsequent behavior towards this partner. The internal representation of such an emotional attitude relies on individual recognition (Aureli & Schaffner, 2002), but does not require a specific memory of who did what and when (‘episodic-like memory’: Crystal, 2009). Perceiving another group member may simply elicit the current emotional attitude associated with that animal. A cognitively more complex process is calculated bookkeeping (required for ‘calculated reciprocity’) (De Waal & Luttrell, 1988; Brosnan & Waal, 2002): individuals keep track of the previous interactions employing mental scorekeeping of both given and received behaviors. Here, reciprocity between two individuals is due to each individual returning the behavior soon after an imbalance between received and given behaviors has been calculated by either individual. In contrast, the agent-based EMO-model suggests that the relatively simple cognitive mechanism of emotional bookkeeping is sufficient (and necessary) for the emergence of reciprocal grooming relationships in particular dyads (Evers et al., 2014; Evers et al., 2015). This implies that the cognitively more demanding process of calculated reciprocity is not required.

The suggestion from the EMO-model that emotional bookkeeping may explain some behavioral patterns in primates and other species is also consistent with empirical data. For instance, a study on male macaques reported that coalition formation in the mating season could be predicted by affiliation patterns several weeks earlier, even after controlling for affiliation in the mating season itself (Berghänel et al., 2011). Female macaques reciprocate grooming to preferred partners over longer time frames (Schino & Aureli, 2009). Similarly, in chimpanzees long-term grooming better predicts reciprocity that short-term grooming (Gomes, Mundry & Boesch, 2009). Moreover, many empirical studies have reported evidence of highly individualized affiliative relationships in animals, where strong social bonds with a few (kin and/or non-kin) partners were often more reciprocal than weak social bonds and could be maintained over long periods of time, up to several years (baboons (Silk, Alberts & Altmann, 2006a; Silk et al., 2010b; Silk et al., 2012), macaques: (Massen & Sterck, 2013); chimpanzees (Langergraber, Mitani & Vigilant, 2009; Lehmann & Boesch, 2009; Mitani, 2009; Koski et al., 2012), African elephants (Archie, Moss & Alberts, 2006; The Amboseli elephants, 2011) and bottlenose dolphins (Connor et al., 2000; Connor, Heithaus & Barre, 2001; Wiszniewski, Lusseau & Möller, 2010; Wiszniewski, Brown & Möller, 2012)). However, these studies cannot completely rule out that the emerging interaction patterns (taken as indicative of the social relationships) result from simple association rules. In short, which cognitive processes are necessary (and sufficient) for variation in relationship quality and long-term social bonds is difficult to investigate in real animals. Agent-based models form a theoretical tool complementary to empirical research to investigate this (Evers et al., 2014; Evers et al., 2015).

Agent-based models (ABM) are a well-established tool to systematically study and understand structuring mechanisms in a complex system of interacting entities (Hogeweg & Hesper, 1979; Bryson, Ando & Lehmann, 2007). The implemented rules and internal (emotional/cognitive) processes of the model entities determine their selection of behaviors (concerning interactions, partner choice and movement), but depend on and in turn affect the (local) social environment, thereby resulting in specific social and spatial structures on the group-level. Agent-based modeling is an ideal tool to compare the effect of different behavioral rules and internal processes, since these are explicitly formulated and their consequences are measurable in a way that is not possible for real group-living individuals. One specific advantage of ABM, especially in the context of studying social interactions of animals in relation to their perceptual, emotional, cognitive and communicative abilities, is the requirement to be explicit, which forces the modeler to focus on these abilities from the point of view of the animal (De Vries & Biesmeijer, 1998).

In a previous paper (Evers et al., 2014), we presented an ABM, dubbed the EMO-model, in which we implemented emotional bookkeeping and its underlying mechanisms. We aimed to base all emotional and behavioral processes in the model on empirical data of despotic macaque species. Moreover, we set parameter values such that mean behavioral rates and durations in the model are within the ranges found in empirical macaque data. Most higher level patterns emerging in the model were found to match empirical data as well, thus strengthening the validity of our model. We consider this EMO-model to be a useful framework for investigating the influence of different key factors on the emergence and maintenance of social relationships. Thus, in a follow-up paper (Evers et al., 2015) we show that ‘choosiness’ is necessary for the emergence of partner-specific reciprocal grooming relationships, given that the modeled individuals possess the capacity of emotional bookkeeping (see also below). In the current paper, we use the EMO-model to investigate how different degrees of ‘choosiness’ and different rates of socio-emotional memory decay influence the stability and duration of grooming relationships.

In the EMO-model, social interactions elicit a positive (satisfaction) or negative emotional response (anxiety) in the individuals, which may affect an individual’s subsequent behavior on the short term. Moreover, individuals in the model assign positive or negative attitudes to their group members, which affect partner-specific behavioral tendencies concerning affiliation (LIKE attitude) and agonism (FEAR attitude) over a longer term (see Fig. T1). In the EMO-model the FEAR attitude is fixed and linked to the difference in (fixed) dominance rank (cf. Bryson, Ando & Lehmann, 2007; Evers et al., 2011; Evers et al., 2012). In contrast, LIKE attitudes are dynamically changing upon (the lack of) affiliation received from group members. The partner-specific LIKE attitude quickly integrates the positive emotional responses to received affiliation from the respective partner and slowly decreases over time. Thus, strong LIKE attitudes may develop fast, but are only maintained towards regular groomers. In this way, dynamic partner-specific LIKE attitudes models a form of emotional bookkeeping (cf. Aureli & Schaffner, 2002; Aureli & Schino, 2004; Schino & Aureli, 2009).

Several studies employing ABMs have explored the effect of choosing particular partners on the emergence of relationships: the model of Campennì & Schino (2014); FriendsWorld (Puga-Gonzalez, Hoscheid & Hemelrijk, 2015); and the EMO-model (Evers et al., 2014; Evers et al., 2015). These models show that reciprocal relationships are found when individuals preferentially direct their behavior to or associate with past interaction partners. Therefore, these models indicate that the features of memory or encounters affect the degree of reciprocity. In the single-generation model of Campenni & Schino the number of memory steps considered is varied. When fewer or no memory steps are considered, so the memory of past events is less prevalent or even absent, no reciprocal behavior is found and relationships are not differentiated. In FriendsWorld, reciprocity is only stronger than in the ‘control’ GrooFiWorld model when aggression has a high intensity, and not when it has a low intensity. Only under conditions of high intensity aggression are encounters differentiated and does reciprocation become differentiated. In both models, selectivity for individuals providing benefits is high. In contrast, our previous investigation using the EMO-model focused on the effects of different degrees of ‘choosiness’ (from absent to low to high) (Evers et al., 2015). It was shown how the emergence of affiliative relationships depend on LIKE-PARTNER SELECTIVTY (LPS), i.e., the degree to which individuals prefer to affiliate with partners that are assigned high LIKE attitudes. When LIKE attitudes did not affect partner choice (LPS = 0), development of affiliative relationships was purely based on proximity and dominance relations. At intermediate partner selectivity (LPS = 0.5 or LPS = 0.9), rank difference was a good predictor of affiliative relationships, i.e., the correlation between rank difference and LIKE attitudes was enhanced. Only high partner selectivity (LPS = 0.95) resulted in the emergence of individualized affiliative relationships that depended on the partner-specific affiliation history (LIKE attitudes). A control-model with fixed LIKE attitudes (based on rank-distance), did not yield such individualized affiliative relationships, even when partner selectivity was high. This shows that, in our EMO-model, dynamical emotional bookkeeping and high partner selectivity are necessary and sufficient for the emergence of individualized affiliative relationships (Evers et al., 2015). In the EMO-model, high partner selectivity allows for a strong coupling of received grooming, developing LIKE attitudes and subsequent grooming partner selection, yielding self-reinforcing affiliative relationships.

The conditions under which emotional bookkeeping can lead to long-term stability of social bonds are still unclear. Whether social bonds are maintained or exist only temporarily may depend on: (a) how often individuals encounter each other; (b) the impact of affiliative behavior on the perceived quality of the relationship (i.e., the LIKE attitude towards the partner); (c) how quickly LIKE attitudes (socio-emotional memory) decay over time; and (d) how selective (‘choosy’) individuals are. In the current paper we specifically explore the effect of the latter two factors: temporal dynamics of emotional bookkeeping and individuals’ ‘choosiness,’ on the establishment of long-term affiliative relationships.

Thus, we investigated in the EMO-model which temporal conditions of emotional bookkeeping are necessary to yield long-term stability of individualized affiliative relationships. To do this, we systematically varied the decay rate of LIKE attitudes, i.e., the timeframe over which the accumulated value of emotional responses to earlier received affiliation is remembered. Additionally, we varied the degree of partner selectivity (LPS), i.e., the degree to which LIKE attitudes affect the individuals’ partner choice and behavior. We investigated which specific settings of *emotional memory decay* and *partner selectivity* would yield long-term social bonds. Particularly, we were interested in the duration, stability and reciprocity of (strong) social bonds, and in how well the internal representation of affiliative relationships (LIKE attitude) corresponded to its expression in externally observable affiliative behavior (grooming).

## Methods

The EMO-model is an Agent-Based Model (ABM), in which model entities possess the same set of behavioral rules but variable internal states, which together regulate the interactions with others in their environment. Internal states are updated after an interaction or, sometimes, e.g., during a grooming bout, almost continuously. In the EMO-model, each model individual has rules that regulate its movements relative to specific others and relative to the whole group. In addition, a model individual has behavioral rules that determine its social interactions with others (action selection). Individuals select probabilistically a behavior from different types of movement (leave, approach, avoid, random walk), affiliation (groom, affiliative signal) and agonism (attack, aggressive or submissive signal). The internal states comprise an emotional state and emotional attitudes towards other individuals. The emotional state of an individual has three components (arousal, anxiety and satisfaction) that are updated on the basis of its own interactions or on observing escalated fights in its vicinity. The arousal component affects the chance that the individual will select an active behavior rather than resting behavior. In addition, based on previous affiliative interactions from an interaction partner, an individual can develop a particular positive assessment (“LIKE attitude”) of this interaction partner. The model individuals also have a fixed FEAR attitude that is based on fixed differences in dominance. In the EMO-model the environment is purely social and only interactions between individuals affect their behaviors, emotional states and LIKE attitudes.

The model is programmed in NetLogo 5.0.2 (Wilenski, 2007). The program code of the model will become available via the *Publications* website of the *Animal Ecology* group (http://www.uu.nl/en/research/animal-ecology/publications). Below, we describe our model according to the updated ODD protocol (Grimm et al., 2010), which is a standardized method of describing agent-based models. This ensures the model description to be more complete and better comparable to other models, allowing also reproducibility of the model. The ODD protocol contains an Overview (1–3), Design concepts (4–11), and Details (12–13). The detailed ODD protocol information on the EMO-model can be found elsewhere (Evers et al., 2014). Additionally, this methods section includes parts on the (14) Simulation experiments and the (15) Statistical analysis, which are not part of the ODD protocol.

### Purpose

The purpose of this model is to explore certain capacities of socio-emotional information integration (emotional bookkeeping) that may be used in macaques to develop and maintain social bonds, and their effect on the emergent properties of affiliative relationships. First, we studied the timeframe over which earlier affiliation (in contrast to current affiliation) is incorporated into LIKE attitudes. In other words, the appreciation of affiliation partners may be based either on a short-term or long-term summary of earlier grooming experiences, and this is expected to affect the structure and stability of affiliative relationships. Second, we varied the partner selectivity, i.e., the degree to which individuals select grooming partners with high LIKE attitudes, to study its effect on the stability and duration of affiliative relationships.

### Entities, State Variables and Scales

We simulated the movements and interactions of 20 macaque-like model individuals. These individuals are characterized by a number of state variables (see Table T1). These concern the dominance strength, waiting time to next interaction, attention to others, the dynamic emotional state and emotional attitudes to others. The last column in Table T1 presents the intended meaning of these individual state variables, which are briefly introduced here and described in more detail in ‘Submodels.’

Individuals are characterized by their dominance strength (myDOM) which ranges from 0.05 (lowest ranking) to 1 (highest ranking individual), which does not change over time or after interactions (Bryson, Ando & Lehmann, 2007; Evers et al., 2011; Evers et al., 2012; Evers et al., 2014). This represents stable dominance ranks, that often last for extended periods and form the typical situation in macaque groups (Bernstein, 1969; Silk, 1988; Rhine, Cox & Costello, 1989; Ostner, Heistermann & Schülke, 2008). Individuals differ in their scheduled time (myTIME), their current scanning probability (myPscan) and in the current width of the view angle (myVIEW˙ANGLE), which change dynamically over the course of the simulation.

Our model entities are further described by their emotional state, consisting of arousal, anxiety and satisfaction (myAROUSAL, myANXIETY and mySATISFACTION), i.e., an individual’s state of alertness and an aversive and a pleasant dimension of the emotional state. Arousal, anxiety and satisfaction change over time depending on the social context the model entity experiences. Model individuals also possess partner-specific emotional attitudes (LIKE and FEAR), which they assign to each other group member. In our model, agonism-related FEAR attitudes are fixed, while affiliation-related LIKE attitudes are dynamically changing over time depending on partner-specific affiliation history (How these emotional states and attitudes change is explained below in ‘Submodels.’).

General model parameters are summarized in Table T2. The modelled environment is a continuous two-dimensional grid (300 × 300 grid units) with a torus shape to exclude disturbing border effects. The length of one grid unit resembles 1 “meter.” We did not explicitly implement ecological features of the environment; in the model an individual’s environment is purely social.

One time step in the simulation resembles 1 MINUTE. One HOUR consists of 60 MINUTES and we defined 12 HOURS as one DAY, 7 DAYS as 1 WEEK and 50 WEEKS as 1 YEAR. To define one MONTH we divided one YEAR (350 DAYS) into 12 equal periods of ca. 29 DAYS. Simulations were run for 504000 time steps, i.e., 2 YEARS, plus a prior stabilization period.

In contrast to earlier papers (Evers et al., 2014; Evers et al., 2015), we here varied two parameters across the simulation experiments, namely the LIKE-HISTORY WEIGHT (LHW) and the LIKE-PARTNER SELECTIVITY (LPS). LHW describes the timeframe over which earlier affiliation (in contrast to current affiliation) is incorporated into the LIKE attitudes and was set to 0, 180, 720, 5400 or 21600 MINUTES, respectively. LPS defines the degree to which individuals selectively prefer valuable individuals as grooming partners and was set to 0.00, 0.50, 0.90, 0.95 or 0.99, respectively (see Table T2). LHW directly affects the speed of the LIKE dynamics, as higher LHW result in a slower decrease and stabilization of LIKE attitudes and other related patterns (see *Submodel ‘LIKE Attitudes’*). Therefore, settings of high LHW required longer stabilization periods prior to the data-recording period of 2 YEARS, than low LHW (see Table T2). Thus, the duration of the stabilization period (TIME_STAB_) was dependent on the setting of LHW and defined as follows:

Note, that the minimum duration of the stabilization period was set to 100 HOURS.

### Process overview and scheduling

Our model is event-driven. Most social behaviors are modelled as discrete events in time, except for moving, resting and grooming, which are modelled as continuous duration behaviors. Time is modelled on a continuous scale and during a simulation run individuals’ activations are regulated by a timing regime. The general process overview is pictured in Fig. F2 and described briefly here. Detailed descriptions of the different emotional and behavioral processes are given below in ‘Submodels.’

Each time, the agent with the lowest schedule time is activated first (see Fig. F2). Whenever an individual is activated, first all model entities update those state variables that may have increased or decreased over the time interval that has passed since the last activation of an entity (arousal, anxiety, satisfaction, LIKE attitudes). If the activated individual had scheduled a movement action, that action is executed; else, ego checks the grouping criteria and, if necessary, employs grouping. If no grouping and no movement are to be performed, ego may select a social behavior or resting or random movement within the group (action selection). Which behavior (and which interaction partner) gets selected depends on ego’s own emotional state, as well as on it’s emotional attitudes towards the potential interaction partners. Subsequently, the selected behavior may affect emotional attitudes of involved individuals, as it may affect the emotional state of ego, of receivers and observers of the behavior. As a consequence, ego, receivers and observers may be activated sooner or later, depending on the behavior executed.

Thus, after activation, the next activation of ego, but also that of interaction partners or bystanders, is scheduled anew. The exact time until an individual’s next activation depends on the behavior performed, received or observed, respectively. As movement, resting and grooming are implemented as duration behaviors, they are performed in bouts. After starting a movement bout ego is activated each 3 s to execute the last movement “step” and to decide whether movement is to be continued. After starting a grooming or resting bout ego is activated anew after 7.5 ± 0.375 min (mean ± SD). Social interactions may involve (and therefore activate) other group members and may also interrupt a grooming or resting bout. Whenever ego receives an attack, it is immediately activated to respond with either fleeing or a counter attack. Whenever ego receives a signal or observes an attack nearby, a fast reaction is required and ego is activated 0.1 ± 0.005 s (mean ± SD) later to select an action.

### Basic principles

In our model, receiving affiliation affects the general emotional state (arousal, anxiety and satisfaction) of individuals, as well as their affiliative attitude (LIKE attitude) directed to the actor of affiliation. In turn, an individual’s emotional state affects its general short-term probability of executing certain behaviors. In this way, emotional processes regulate spontaneous behaviors as well as appropriate responses to received behaviors. Partner-specific LIKE attitudes, which summarize earlier received affiliation from specific individuals on a shorter- or longer-term, depending on the setting of LHW (LIKE-HISTORY WEIGHT) in the model. This models differences in decay rate of the memory of affiliative interactions. LIKE attitudes in turn affect the probability of affiliating with these individuals and, in this way, regulate the development and maintenance of affiliative relations via LPS (LIKE-PARTNER SELECTIVITY). This ranges from no use of the LIKE-attitude (LPS = 0), representing a lack of emotional bookkeeping, to the strong directing of partner selection based on LIKE attitudes (LPS = 0.99). Varying the settings of LHW and LPS yields insights on the stability and duration of individual-specific affiliative relationships.

### Emergence

In agent-based models, individual behavior is imposed by the model rules, while group-level properties are usually not implemented explicitly into the model, but rather emerge from the interactions of the lower-level entities, i.e., the individuals. In our model, behavioral patterns, e.g., affiliation, aggression and proximity, are emergent properties arising from the interactions of the model entities. The structure of the network of LIKE attitudes and group level properties such as reciprocity and partner-specificity are an emergent property arising from the interrelation between emotional attitudes and affiliative behavior.

### Adaptation

The model entities change their behavior in response to changes in their general emotional state (see submodels Aurosal, Anxiety, Satisfaction) and their emotional attitudes towards others (see submodel LIKE Attitudes) and individuals (implicitly) seek to increase satisfaction and to decrease anxiety. As appropriate behavior is mediated by emotional processes, this yields a homeostatic regulation system. In this way, we aimed to produce adaptive (in the sense of flexible) behavior and emerging group properties that are representative of observations of the social behavior of real primates.

### Learning

Individuals in the dynamic attitude model regularly update their partner-specific LIKE attitudes assigned to other group members, based on earlier grooming received from these individuals. This may be seen as a (basic) form of learning. The LIKE attitude represents the memory of the summarized positive emotion elicited by affiliative behavior received from a particular individual. This is similar to a learning process with reinforcement of the LIKE-attitude after receiving affiliative behavior from a particular individual and fading of the LIKE-attitude when no affiliative behavior from this particular individual is received. Emotional bookkeeping thus provides individuals with information on valuable affiliation partners, which may dynamically change over time according to these partners’ behavior. See the section on LIKE attitudes below for a detailed description of how LIKE attitudes are updated during bouts of grooming and during periods of non-grooming.

### Sensing

Individuals in our model may perceive the location, certain behaviors and signals of other group members, but only ‘locally’ within certain distances and within a specific view angle (see submodels Perception, Scanning). The exact distances depend on the salience of the perceivable information. Individuals are able to perceive (or know) the dominance strength of other group members and perception of a group member elicits ego’s internal valuation of this group member, i.e., its FEAR and LIKE attitude that are assigned to this specific individual.

### Interactions

Social interactions in our model can be categorized as affiliative, submissive and aggressive behaviors. Affiliation comprises grooming, affiliative signalling and approaching; submission comprises leaving, submissive signalling and avoiding; and aggression comprises attacking and aggressive signalling. Interactions are implemented identically as described in the introductory paper on the EMO-model (Evers et al., 2014).

Potential interaction partners are the 10 nearest recognizable individuals (within MAX˙DIST and ego’s current view angle). The potential behavioral probabilities towards these 10 (or less) individuals depend on ego’s emotional attitudes (FEAR and LIKE) assigned to those individuals and ego’s general emotional state (arousal, anxiety, satisfaction). One behavior towards one specific interaction partner is chosen randomly according to those probabilities.

Some social interactions can only be performed towards group members within a certain distance. Individuals within INTERACT˙DIST (1 m) can be groomed, left or attacked. Individuals within PERS˙DIST (5 m) can receive (affiliative, submissive or aggressive) signals. Individuals within MAX˙DIST (50 m) can be approached and individuals within PERS˙DIST (5 m) can be avoided.

### Stochasticity

In our model, many processes are not implemented deterministically, but include some degree of stochasticity, to produce variability in those processes. Those processes include action selection, the determination of the winner of an escalated fight, the random walk procedure and the timing regime.

### Observation

For the analysis of our model, we only used data that were recorded during the last 504.000 time steps of each simulation run, i.e., the 2 YEARS after the stabilization phase, to avoid transient spatial and social group effects due to the initial random placement.

Dyadic LIKE attitudes were sampled every 3.5 DAYS and then averaged (per dyad) over the last YEAR for each simulation run. Dyadic grooming rates were recorded (per dyad) over each recording interval of 3.5 DAYS and then divided by the duration of the recording interval to obtain average hourly behavioral rates.

Note that LIKE attitudes and grooming are always directed from an actor to a receiver and are thus not symmetric by definition.

To illustrate the variation in LIKE attitudes over time, we also provide snapshots of the LIKE attitudes at single points in time (Figs. F3 and F4). For this snapshot, LIKE attitudes were sampled at the time step after the first and the second YEAR of the recording period.

To calculate long-term measures, such as the number of preferred partners, the bout duration and the total duration of grooming partner preferences, grooming rates were sampled over the whole recording period, i.e., over 2 YEARS.

### Initialization

The initial settings are summarized in Table T1 (cf. Evers et al., 2014). At the initialization of each simulation run, the *x*-coordinates and the *y*-coordinates of the 20 individuals were drawn randomly from a predefined circular sphere with an arbitrary diameter of 50 m. The individual’s initial heading was set to a random orientation between 1° and 360° and the initial view angle was set at 120° for each individual. Each individual’s level of arousal was set to the default arousal level (0.09) and the level of anxiety and satisfaction was set to 0.0. LIKE attitudes were initialized at 0.0. The initial schedule time for each individual was drawn randomly from a normal distribution with a mean of 1 min and a standard deviation of 0.05 min.

### Submodels

This section describes the main processes implemented in the EMO-model (cf Evers et al., 2014), as far as needed for an understanding of the current paper. It covers the implementation of the processes regulating the emotional state (arousal, anxiety and satisfaction) and the partner-specific emotional attitudes (FEAR and LIKE), the processes of action selection, perception, scanning, movement, grouping, grooming and resting, and attack, counter-attack and escalated fight. For a detailed description of the model, the substantiation of the modelled emotional processes and behavioral rules using empirical data, and parameterization and validation of our model we refer the interested reader to our earlier paper (Evers et al., 2014).

#### Arousal

In our model, an individual’s arousal level, i.e., its responsiveness or tendency to be active (cf. Mason, 1973; Winslow, Newman & Insel, 1989; Jones, 2003; Rendall, 2003; Paul, Harding & Mendl, 2005), changes on the basis of encountered and observed social interactions. The extent of arousal change depends on the emotional salience of the stimulus (Table T3). Arousal level was scaled between 0 (inactive) and 1 (highly aroused), with 0.09 being the default level (Table T1). Arousal increases in response to receiving, executing or observing aggression or when in proximity of a dominant individual. The specific arousal change depends on the stimulus, for example receiving an attack increases arousal more than observing an escalated fight (Table T3). Arousal increases per event and the maximum arousal level is 1. On the other hand, arousal may also decrease over time. In response to receiving submissive or affiliative behavior and when executing affiliative behavior it may decrease below the default level of 0.09. When the individual is in a lasting social context, such as being groomed or in proximity of a dominant individual, arousal decreases or increases with a default rate of 0.02/min to the limit value corresponding to this social context (see myAROUSAL˙LIMIT in Table T1). When this social context ends, the arousal level approaches with a rate of 0.02/min the default level of 0.09. A high arousal level increases the probability to perform active behaviors (any behavior except resting) and the probability to employ social vigilance, i.e., scanning behavior. (How we arrived at this implementation of arousal in our model is explained in detail in supplementary material to Evers et al. (2014) (journal.pone.0087955.s006.DOC), which also includes the references to the empirical literature on which this is based.)

#### Anxiety

In our model, anxiety level, i.e., an individual’s general ‘fearfulness’ in response to negative stimuli within the current social environment, was scaled between 0 (not anxious) and 1 (highly anxious). Anxiety increases immediately (with different degrees: see Table T3) in response to aggression, i.e., ‘Receiving an attack’ (cf. macaques: Aureli & van Schaik, 1991; review Aureli & Schaffner, 2002; chimpanzees: Wittig et al., 2015) or ‘Giving an attack’ (cf. macaques: Das, Penke & van Hooff, 1998; chimpanzees: Wittig et al., 2015), ‘Receiving an aggressive signal’, ‘Losing an (escalated) fight’ or ‘Observing an escalated fight nearby.’ It decreases instantaneously in response to winning a fight, receiving submission or an affiliative signal. Anxiety decreases over time towards 0 when giving (rate: 0.01/min) or receiving (rate: 0.02/min) grooming (cf. baboons: Wittig et al., 2008). When none of these behaviors occur, anxiety decreases with a rate of 0.002/min to the default level of 0. High anxiety levels increase probabilities of affiliation and submission and decrease probabilities of aggressive behaviors.

#### Satisfaction

In our model, satisfaction level, i.e., an individual’s general ‘contentedness’ in response to positive stimuli within the current social environment, was scaled between 0 (not satisfied) and 1 (highly satisfied). There are two limit values (0 and 1), towards which the satisfaction level can change over time, depending on the behavioral context of the individual. Satisfaction increases towards the limit value 1 when receiving grooming (rate: 0.1/min)(GR˙SAT˙INC) or when giving grooming (rate: 0.05/min)(GG˙SAT˙INC)(Boccia, Reite & Laudenslager, 1989; Gust et al., 1993; Aureli, Preston & De Waal, 1999; Shutt et al., 2007). Without grooming, satisfaction decreases over time with a constant rate (0.02/min)(DEF˙SAT˙DEC) towards the default value 0 (Tables T1 and T3). High satisfaction decreases the probability to select affiliative behavior.

#### FEAR attitudes

In our model, individuals assign a partner-specific FEAR attitude to each group member. FEAR attitudes resemble the difference in dominance strength between an individual (*i*) and another group member (*j*) and are calculated as FEAR*ij* = myDOM*j* − myDOM*i*, i.e., ranging from −0.95 to +0.95. Thus, FEAR attitudes are directional and not symmetric. Based on the fixed dominance strengths (myDOM), FEAR attitudes are fixed over the course of our simulation and are, thus, not affected by social interactions. Yet, they do affect the individual’s valuation of its potential aggression risk related to the respective group member. High FEAR attitudes result in decreased probabilities of aggression (i.e., attack, aggressive signal) and increased probabilities of submission (i.e., leaving, submissive signal, avoidance) towards the respective group member.

#### LIKE attitudes

LIKE_*ij*_ describes individual *i*’s affiliative valuation of a specific group member j. LIKE attitudes may have values ranging from 0 (neutrally valued affiliation partner) to 1 (highly valued affiliation partner). LIKE attitudes are not symmetric per se, as they are dynamically updated dependent on earlier affiliation received from specific individuals. A high LIKE attitude affects ego’s judgement of the affiliative ‘value’ of the respective group member and results in increased probabilities of affiliation (i.e., grooming, affiliative signalling and approaching) towards this specific group member.

LIKE attitudes are dynamically updated upon receiving grooming. The exact increase of LIKE_*ij*_ depends on individual *i*’s current increase in satisfaction in response to grooming time received exclusively from individual *j*. The current satisfaction level of *i* due to the grooming received from partner *j* is represented by the partner-specific variable PARTNER˙SAT_*ij*_, which increases during the grooming with a rate of 0.1/min (GR˙SAT˙INC). The LIKE attitude from *i* to *j* is updated by combining the value of the last LIKE_*ij*_ with the current satisfaction level PARTNER˙SAT_*ij*_, as follows:

\begin{eqnarray*} {\text{LIKE}}_{i j}({t}_{n})=\max \left\{\begin{array}{} \displaystyle \frac{\mathrm{LHW}\ast {\text{LIKE}}_{i j}({t}_{n-1})+({t}_{n}-{t}_{n-1})\ast {\text{PARTNER_SAT}}_{i j}({t}_{n})}{\text{LHW}+({t}_{n}-{t}_{n-1})}\\ \displaystyle {\text{PARTNER_SAT}}_{i j}({t}_{n-1}) \end{array}.\right. \end{eqnarray*}

Here, *t_n_* is the current time, *t*_*n*−1_ is the time of the last update and (*t_n_* − *t*_*n*−1_) is the time since the last update (in MINUTES). LIKE_*ij*_(*t_n_*) is the updated (new) value of the LIKE attitude from *i* to *j* and LIKE*ij*(*t*_*n*−1_) is the former level of LIKE. LHW (LIKE-HISTORY WEIGHT) weighs how strongly the updated LIKE attitude depends on earlier (emotional responses to) affiliation history as opposed to recent (emotional responses to) affiliation. The value of the parameter LHW differs per simulation run.

**Figure 1:**
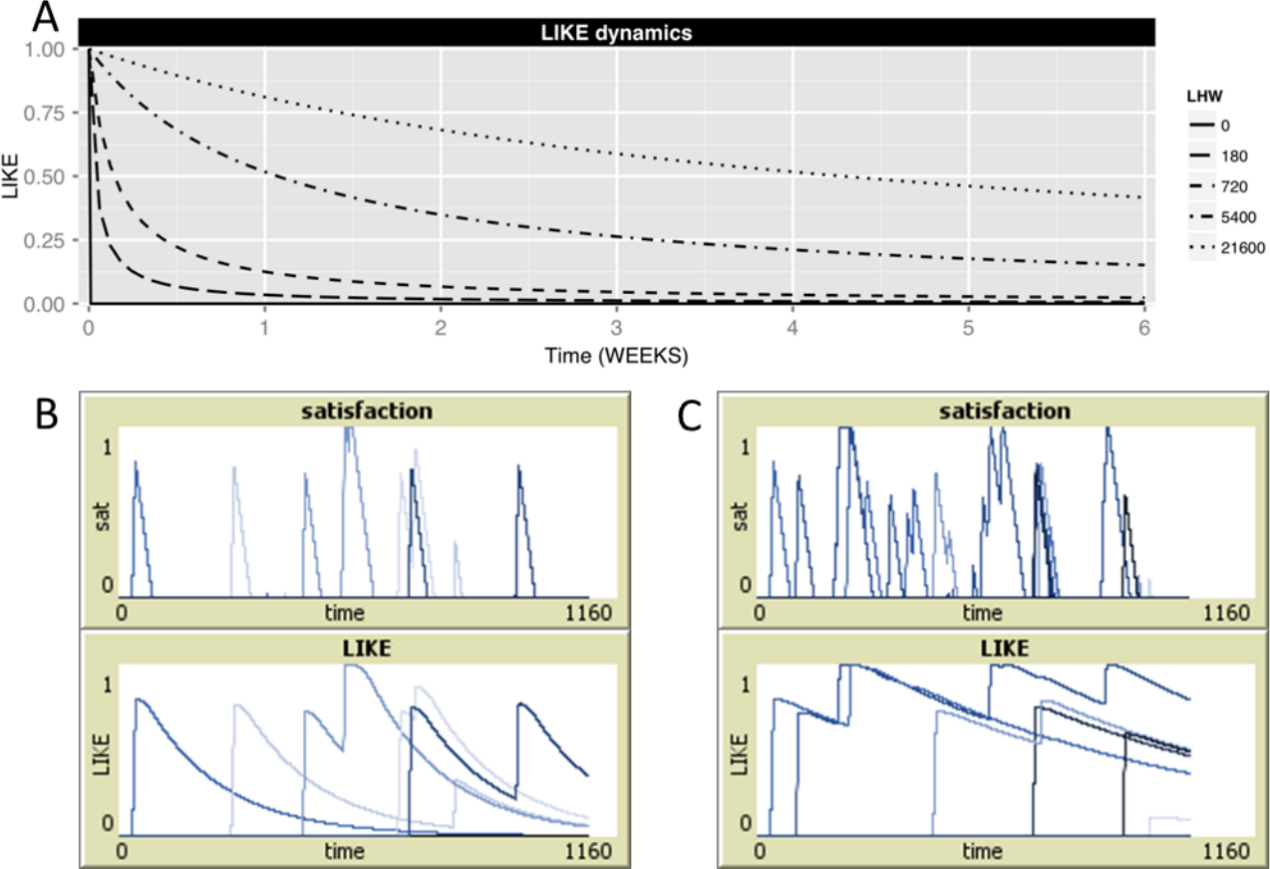
Satisfaction and LIKE dynamics. (A) Shows the decrease of the level of LIKE_*ij*_, starting from initial value of 1.0, for different settings of LHW (solid line: LHW = 0, long-dashed line: LHW = 180 (half-life = 3 HOURs), dashed line: LHW = 720 (half-life = 1 DAY), dot-dashed line: LHW = 5,400 (half-life = 1 WEEK), dotted line: LHW = 21,600 (half-life = 1 MONTH)), given that individuals *i* and *j* do not have affiliative interactions during the next 6 weeks. Higher LHW imply a slower decrease of LIKE, a higher half-life of LIKE and, thus, slower LIKE dynamics. Note, that when LHW = 0, LIKE_*ij*_ decreases very quickly from 1.0 to 0.0 (within 50 MINUTES). (B, C) show two examples of partner-specific satisfaction and LIKE levels in one individual *i* due to grooming bouts received from five different partners *j* (B: LHW = 180, C: LHW = 720). Each partner is depicted in a different shade of blue.

Thus, the updated LIKE attitude (LIKE_*ij*_(*t_n_*)) combines the current level of satisfaction associated with the partner (PARTNER˙SAT_*ij*_(*t_n_*)) with the recent history of partner-specific satisfaction levels summarized in the former level LIKE_*ij*_(*t*_*n*−1_) (upper line in the above equation). In this updated LIKE attitude, the former level of LIKE (LIKE_*ij*_(*t*_*n*−1_)) and the current level of satisfaction associated with the partner (PARTNER˙SAT_*ij*_(*t_n_*)) are weighted by LHW and (*t_n_* − *t*_*n*−1_), respectively. The higher the value of LHW, the less the updated LIKE attitudes are influenced by the current satisfaction level. When, however, the current satisfaction level PARTNER˙SAT_*ij*_(*t_n_*) exceeds this new LIKE value, LIKE_*ij*_(*t_n_*) is set to the level of PARTNER˙SAT_*ij*_, ensuring that LIKE attitudes are always higher than or equal to the current partner-specific satisfaction level (lower line in the above equation). Figures 1B and 1C, shows examples of the dynamically changing levels of partner specific satisfaction and LIKE for LHW = 180 (Fig. 1B) and LHW = 720 (Fig. 1C). Note that, while PARTNER˙SAT_*ij*_ decreases quickly after being groomed, this satisfaction is integrated in the LIKE attitude, which is retained in memory and only slowly decays over time, with the rate of decay depending on the value of LHW (Figs. 1B and 1C). Note also, that when LHW = 0 no earlier affiliation is retained in memory and LIKE_*ij*_ thus simply equals the value of PARTNER˙SAT_*ij*_.

Whenever PARTNER˙SAT_*ij*_ = 0, i.e., no affiliative behavior was received recently from the respective partner, LHW can be seen as the half-life of LIKE attitudes, which determines the time it takes before LIKE attitudes decrease to half of their initial value. For example, when LHW = 180, it takes 180 MINUTES (3 HOURS) for LIKE_*ij*_ to decrease from 1.0 to 0.5. Higher LHW results in a longer half-life, i.e., in slower dynamics, of LIKE attitudes (see Fig. 1, upper panel). Note, that at LHW = 0 (i.e., LIKE_*ij*_ = PARTNER ˙SAT_*ij*_), LIKE decreases equally fast as satisfaction levels. Note further, that the quick increase of LIKE attitudes during receiving grooming is not dependent on the setting of LHW.

In sum, current affiliation received from a partner may quickly increase LIKE and/or maintain a high level of LIKE, while the lack of current affiliation will result in a slowly decreasing LIKE attitude. How slow LIKE attitudes decrease in the absence of received grooming, depends on the setting of LHW. Thus, LHW in combination with the frequency and duration of grooming interactions eliciting satisfaction (PARTNER˙SAT_*ij*_) determine how long a high LIKE attitude is maintained.

#### Action selection

In our model, activated individuals may select one of various possible actions. These actions may be directed to other individuals or may involve resting or random movement within the group.

The probability to execute a specific behavior towards another group member depends on (a) the distance of the individual to ego, (b) ego’s emotional state (arousal, anxiety and satisfaction), (c) ego’s FEAR and LIKE attitudes assigned to the potential interaction partners and (d) the parameter setting of LIKE-PARTNER SELECTIVITY (LPS), i.e., the degree to which high LIKE attitudes are important for the selection of affiliation partners. The emotional state facilitates behavior that is appropriate to the individual’s position and situation within the social group in general, while emotional attitudes facilitate behavior that is appropriate towards specific group members.

First, the 10 (or less) potential interaction partners are determined. Then the possible behaviors towards each of these individuals are determined dependent on their distance to ego. Finally, the probabilities for those possible behaviors towards each potential interaction partner are calculated. According to these probabilities, one of the possible behavior-partner combinations is randomly selected and executed. The details on the calculation of the probabilities for affiliation, aggression, submission and avoidance are described in Supplemental Information 3 and are briefly summarized here.

Ego’s probability to direct affiliation towards individual *j* increases with increased LIKE_*ij*_ (given that LPS > 0) and with increased intrinsic affiliation motivation of ego, i.e., with higher satisfaction or lower anxiety level. At LPS = 0, the level of LIKE_*ij*_ has no effect on the affiliation probability. With higher LPS the probability to choose *j* rather than *k* as grooming partner depends more strongly on the difference between LIKE_*ij*_ and LIKE_*ik*_ (see the steeper slopes of the lines at increased LPS values in Fig. 1A. Ego’s probability to direct aggression, i.e., attacking and aggressive signalling, towards individual *j* decreases with increased FEAR_*ij*_. Moreover, increased anxiety results in the aggression probability to be more conservative or risk avoiding. Ego’s probability to direct submissive behaviors towards individual *j* increases with increased FEAR_*ij*_ and with increased anxiety (see Fig. 1B).

#### Perception

Individuals in our model can individually recognize other group members within a maximum perceivable distance of 50 m (MAX˙DIST) and within the currently employed view angle. Group members are always perceived within 1 m (INTERACT˙DIST). The view angle is by default 120° (VIEW˙ANGLE) or else 360° (MAX˙ANGLE) when ego is scanning. Model entities can judge whether at least three other group members are present within 20 m (NEAR˙DIST) and within the currently employed view angle. Furthermore, individuals in our model are capable to judge whether their distance to the furthest group member exceeds 100 m (FAR˙DIST). The two latter criteria are used by ego to decide whether grouping behavior should be executed.

Individuals can also perceive signals, which were directed at them from others within 5 m (PERS˙DIST) when in ego’s view angle. Individuals orient to their interaction partner and orient towards an escalated fight. In these situations, if ego or one of the interaction partners was scanning, scanning was stopped.

#### Scanning

When employing scanning behavior, an individual is turning its head right and left, thus expanding its view angle to 360°(MAX˙ANGLE) instead of the default view angle of 120°(VIEW˙ANGLE). Individuals can only employ scanning when resting, and not during moving bouts or social interactions. The probability to perform scanning behavior increases with ego’s current arousal level (see Table F2).

#### Movement

Concerning movement behavior, individuals in our model may either move towards or from other group members (approaching, grouping, fleeing, leaving and avoiding) or they may execute random movement within the group. Movement behavior in our model takes time and is implemented as movement bouts with a constant speed (0.6 m/s), which is reasonable for macaques (Beisner & Isbell, 2009). During such a movement bout, movement is executed step by step. After starting a movement bout, ego is activated each 3 SECONDS to execute the movement it was to perform during this time interval and to decide whether movement is to be continued (see also Fig. F2).

After ending a movement bout ego always performs a proximity update. Ego checks whether any individuals towards which it directs a (high) FEAR attitude (i.e., higher-ranking group members) are now (or still) nearer than 5 m (PERS˙DIST), as this will update the arousal level ego approaches over time (myAROUSAL˙LIMIT) (Table T1). Additionally, other individuals who have (high) FEAR attitudes towards ego are updated on ego’s new spatial location, which may in turn affect the level their arousal will approach over time.

#### Grouping

Before selecting a social behavior, model entities always check whether grouping should be executed. Grouping will be selected if less than three (MIN˙OTHERS) group members are located within 20 m (GROUP˙DIST) and 360°(MAX˙ANGLE) or whenever any group member is further away from ego than 100 m (FAR˙DIST). When grouping is to be performed, ego simply approaches any randomly selected group member.

#### Grooming and resting

In our model, grooming and resting behavior are implemented as duration behaviors, which are executed in bouts. When starting a grooming or resting bout, ego’s next activation is scheduled several minutes later to choose its new behavior. Ego may be disturbed and activated earlier in response to receiving an attack or a signal, or after observing an escalated fight nearby. The average schedule time for grooming was set at 7.5 ± 0.375 min (mean ± SD). This resulted in average grooming durations of 5.7 to 6.3 min (Evers et al., 2014). Grooming affects the arousal level, satisfaction and LIKE-attitude of the groomed individual.

#### Attack, counter-attack and ecalated fight

Upon receiving an attack the respective model individual is immediately activated to respond with either fleeing or a counter-attack. This immediately increases the anxiety level of the attacked individual. The probability to respond with a counter-attack depends on the rank distance between the individuals, where an attack from a lower-ranking individual enhances the probability of a counter-attack, while an attack from a similar- or higher-ranking individual reduces the probability of a counter-attack. When a counter-attack was selected in response to an attack, we call this an escalated fight. The winner and loser of such a fight are determined randomly according to the individuals’ win chance *w_ij_* = 1∕(1 + exp(−*η*∗(myDOM_*i*_ − myDOM_*j*_))), where *η* determines the slope of the sigmoidal win chance curve (De Vries, 2009). When no counter-attack is selected in response to an attack, the attacked individual is defined as the loser and the attacker as the winner of this aggressive interaction. After an attack or an escalated fight, the loser flees from the winner, while the winner is scheduled anew shortly after. Whenever an escalated fight takes place, individuals nearby get activated and their arousal level gets increased. Moreover, these individuals are activated shortly after to enable an appropriate reaction in response to the event.

### Simulation experiments

In this paper we present the results of different settings of the *EMO-model*, in which individuals dynamically update their LIKE attitudes according to earlier received affiliation by specific group members and subsequently use these LIKE attitudes to choose affiliation partners.

We examined five different settings of LPS, i.e., LPS was set to either 0.00, 0.50, 0.90, 0.95 or 0.99, respectively. LPS described the degree to which individuals preferred to affiliate with individuals towards which they directed high LIKE attitudes.

We also examined five different settings for LHW, i.e., LHW was set to 0, 180, 720, 5,400 or 21,600 MINUTES, respectively. LHW described the speed of the LIKE dynamics in our model, where higher LHW would result in a slower decrease of LIKE attitudes over time.

For this paper we examined all 25 combinations of the different parameter settings of LPS and LHW.

LPS = 0.0 resembles a special null model setting. Here, individuals have no preference to select specific affiliative partners concerning LIKE attitudes whatsoever. In other words, individuals do not use LIKE attitudes during affiliative partner selection. Therefore, the behavioral rates in the null model setting should yield the same patterns for all settings of LHW. The null model setting serves as a control setting to assess the effect of the presence of any affiliative partner preference based on emotional bookkeeping.

For each combination of LHW and LPS, 10 independent simulations were run, resulting in a total of 250 independent simulation runs.

### Statistical analysis

We first explain how specific summarized measures were defined and calculated from the recorded data. We continue with the statistics that were used to compare the properties of different subgroups or individual categories. Finally, we explain how we calculated specific statistics, i.e., the row-wise Pearson correlation coefficients and group-level reciprocity. All statistical analyses were performed in R 2.15.2 (R Core Team, 2012).

Individual values of LIKE attitudes and grooming rates were calculated as the sum of all dyadic LIKE attitudes and grooming rates that an individual directed to others. Group means of grooming rates were calculated as the mean of all individual behavioral rates.

We further determined the preferred grooming partners of ego, based on their relative relevance to ego. To do this, we calculated the percentage of grooming an individual gave to each partner per MONTH, based on the total amount of grooming this individual gave to others during this MONTH. Those partners, which received more than 10% of an individual’s total amount of grooming, were called *preferred partners* (cf. “primary partners” Barrett et al., 1999). In this way, the preference for specific partners over other partners is independent of the total amount of grooming given. The number of an individual’s *preferred partners* was counted per MONTH and averaged over the total recording period of 2 YEARS.

A bout of grooming partner preference was defined as a period of consecutive months for which a specific individual remained a preferred partner of ego. To calculate the bout duration of grooming partner preferences, we simply determined the average duration (in MONTHS) of all bouts of grooming partner preference of a dyad over the total recording period of two YEARS. To calculate the total duration of grooming partner preference, we determined the sum of all bout durations of grooming partner preference of a dyad over the total recording period of two YEARS.

To examine the relation between LIKE attitudes and grooming rates, we calculated the row-wise Pearson correlation coefficients (De Vries, 1993) between the dyadic grooming rates and the dyadic LIKE attitudes, using the grooming rates and LIKE attitudes averaged over one year. We calculated the correlation coefficients per simulation run and then averaged them over all runs per setting using a Fisher-z-transformation.

To assess the reciprocity of grooming partner preferences at the group level we calculated the Kendall’s tau row-wise matrix correlation between the matrix of the total durations of grooming partner preferences over two YEARS and its transposed matrix (Hemelrijk, 1990; De Vries, 1993) using the R software package DyaDA (Leiva et al., 2010).

## Results

### General patterns of LIKE attitudes and grooming

#### Distribution of LIKE attitudes

**Figure 2:**
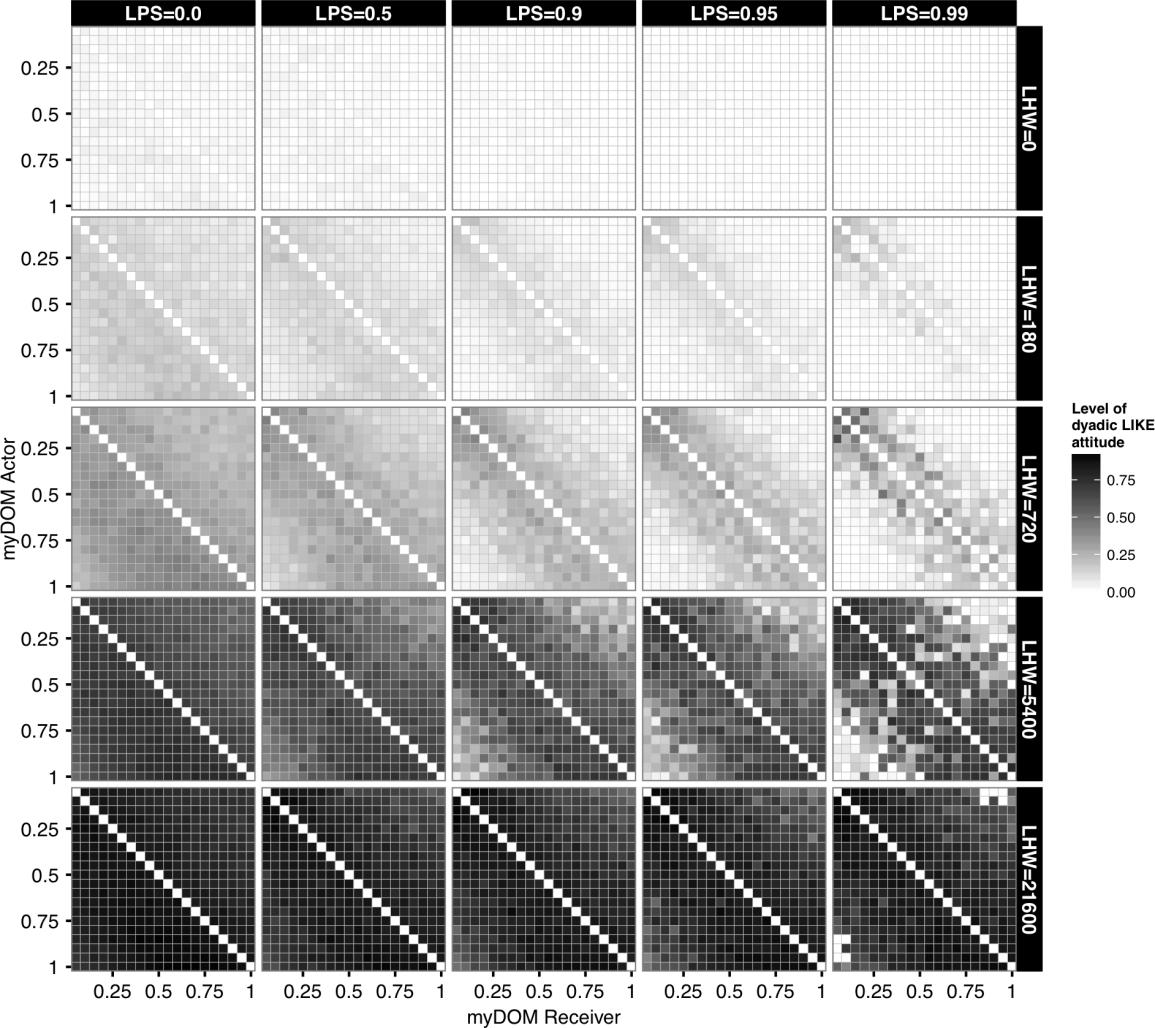
Average LIKE attitudes. This figure shows the distribution of LIKE attitudes among the individuals of a group for different settings of partner selectivity (LPS) and LIKE dynamics (LHW). Higher LPS defines a higher preference for affiliation partners with high LIKE attitudes. Higher LHW (Like history weight) defines slower LIKE dynamics. LIKE attitudes are directed from actors (*y*-axis) to receivers (*x*-axis), both are ordered by dominance strength, ranging from low-ranking (myDOM = 0.05) to high-ranking (myDOM = 1.00). The plot shows the LIKE attitudes of one example run, averaged over one YEAR. Dark shades represent high LIKE attitudes. Values at the diagonal are by definition not applicable.

First, we are interested in how the pattern of developed LIKE attitudes within the group depended on the different settings in our model, i.e., the half-life of the LIKE attitudes (LHW: the LIKE-HISTORY WEIGHT explained in Fig. 1) and the selectivity of individuals when choosing affiliation partners (LPS). In Fig. 2 the level of the LIKE attitudes, averaged over one YEAR, between all individuals of one example run are plotted. A large difference in the average level of LIKE attitudes is visible for the different settings of LHW, which determines the speed of the LIKE dynamics over time. At higher LHW, i.e., slower LIKE dynamics due to long memory, the average value of LIKE attitudes that developed towards others was higher (Fig. 2).

This can be understood as follows. At high LHW the LIKE attitudes decrease very slowly over time. Therefore, high levels of LIKE attitudes are easily (and often even based on only a few interactions) maintained. When LHW was given the extreme value of 21,600 (implying a half-life of one MONTH), we found almost no differentiation in average LIKE attitudes among the dyads (Fig. 2, bottom row matrices). Individuals developed high LIKE attitudes to almost all other group members and these attitudes only decreased very slowly in time. As a consequence, all LIKE attitudes were approximately equally high for all dyads. At the other extreme, when LHW = 0, the average value of LIKE attitudes was close to zero (Fig. 2, top row matrices). Here, emotions elicited by earlier events were not incorporated when updating the LIKE attitude and only very recent affiliative behavior (which elicited a certain degree of temporary satisfaction, PARTNER˙SAT) affected affiliative partner choice. Therefore, LIKE attitudes hardly developed and could not be maintained on a longer term. This resulted in very low average LIKE attitudes.

**Figure 3:**
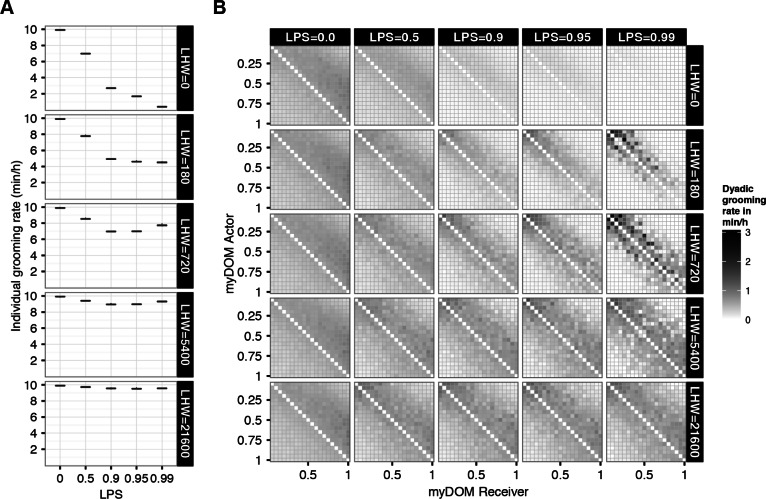
Grooming rates. (A) This figure shows the group mean of the individual grooming rates for different settings of selectivity (LPS) and LIKE dynamics (LHW). The box-plots show individual grooming rates (in minutes per hour) of 10 simulation runs, averaged over one YEAR. (B) This figure shows the distribution of the dyadic grooming rates within the group for different settings of selectivity (LPS) and LIKE dynamics (LHW). Grooming is directed from actors (*y*-axis) to receivers (*x*-axis), both are ordered by dominance strength, ranging from low-ranking (myDOM = 0.05) to high-ranking (myDOM = 1.00) individuals. The plot shows the grooming rates of one example run (the same run as in Fig. 2) in minutes per hour, averaged over one YEAR. Dark shades represent high grooming rates. Values at the diagonal are by definition not applicable.

Due to the very fast or very slow changing LIKE attitudes at low and high LHW, the setting of partner selectivity (LPS) had almost no effect on the distribution of LIKE attitudes (see also further below). This was different at intermediate LHW settings (LHW = 180, 720 or 5,400, implying a half-life of 3 HOURS, one DAY and one WEEK, respectively). Here, in the null model setting (LPS = 0), where individuals selected affiliation partners irrespective of their LIKE attitudes, LIKE attitudes were relatively equally distributed among all group members (Fig. 2, first matrix in rows 2, 3 and 4). So, although earlier affiliative episodes were incorporated in the LIKE attitudes, due to the absence of affiliative partner selectivity, individuals engaged with others purely on the basis of proximity and dominance rank. This resulted in slightly higher LIKE attitudes directed down the hierarchy (i.e., the lower triangular half of the matrices), as grooming was more directed up the hierarchy in this setting (see also Fig. 3B). At higher LPS, individuals chose affiliation partners more selectively, basing their choice more on their LIKE attitudes. As a result, average LIKE attitudes got more symmetric and more differentiated within the group (Fig. 2, rows 2, 3 and 4). Moreover, LIKE attitudes towards similar-ranked partners were higher than LIKE attitudes towards distant-ranked partners. When LPS ≥ 0.95, average LIKE attitudes got differentiated also within subsets of similar-ranked dyads, and were not merely based on rank-distance, but (also) on a strong feedback between partner choice and the LIKE updates, and therefore became partner-specific (Evers et al., 2015).

Note, that in Fig. 2 each LIKE attitude from *i* to *j* is the average over one YEAR and therefore summarizes all LIKE relationships from *i* to *j* that developed (and perhaps also vanished again) over the whole period of one YEAR. To also illustrate the dynamics of the LIKE attitudes over time, we compared the averaged LIKE attitudes of Fig. 2 with snapshots of the LIKE attitudes at a single point in time, i.e., after the first and after the second YEAR of the total recording period of the same simulation run (see Figs. F3 and F4).

From the LIKE snapshot plots, we noticed that at intermediate LHW settings and in the absence of partner selectivity (i.e., null model setting: LPS = 0), individuals developed high LIKE attitudes towards only a few individuals simultaneously, strongly suggesting that they regularly switched partners in an almost random fashion over time (compare first matrix of intermediate panels in Figs. F3 and F4). As a result, the averaged LIKE attitudes were almost equally distributed over the whole group (Fig. 2, first matrix of intermediate panels). In contrast, when individuals were more selective (LPS ≥ 0.9), individuals still switched partners, but potential partners were now more restricted to the more similar-ranking individuals (compare Figs. F3, F4 and Fig. 2, last matrix of intermediate panels).

To summarize, how long LIKE attitudes were maintained over time and also the within-group variation of LIKE attitudes, depended on the specific settings of LHW and LPS. Increased LHW resulted in LIKE attitudes decreasing slower over time. Only at intermediate LHW and high LPS, LIKE attitudes were strongly qualitatively differentiated within the group (e.g., see last matrix in third row of Fig. 2).

#### Distribution of grooming

Above, we saw that higher LHW results in higher average LIKE attitudes for all group members, as LIKE attitudes decreased slower over time. LIKE attitudes, i.e., the internal representation of affiliative partner appraisal, are difficult to examine in real animals. Usually, researchers try to derive this internal representation from externally measurable behaviors such as for example the amount of grooming given to others (e.g., Barrett & Henzi, 2002; Crockford et al., 2013). Therefore, we were interested in how the distribution of LIKE attitudes in our model affected the distribution of grooming given to others in our model.

We first examined the average grooming rates per setting. In general, at higher partner selectivity (LPS) grooming rates were decreased and this decrease was stronger at faster LIKE dynamics (i.e., lower LHW) (Fig. 3A). High selectivity (LPS) resulted in more selective grooming towards individuals with high LIKE attitudes, and therefore in less grooming directed to individuals with low LIKE attitudes. As a result, the total grooming rate generally decreased. Note however, that for LHW = 720 or 5,400 average grooming rate increases again at higher LPS.

To explain the effect of LPS and LHW on grooming rates in more detail, we examined the distribution of dyadic grooming within the group. First, for the null model setting (LPS = 0, first column in Fig. 3B) there is no affiliative partner selectivity, and therefore grooming distributions were similar across all settings of LHW. This can easily be understood: since individuals do not use their LIKE attitudes when choosing grooming partners, the dynamics of the LIKE attitudes does not have an effect on the individuals’ behavior. At LPS = 0, more grooming was directed up the hierarchy than down the hierarchy, and slightly more grooming was directed towards similar-ranking partners than towards distant-ranking partners (first column in Fig. 3B).

Next, for the LHW = 0 setting (top row in Fig. 3B), that is, when the LIKE attitude from emotions elicited by earlier received affiliation was not retained, individuals maintained high LIKE attitudes towards others only for very brief periods. At increased partner selectivity, individuals are more ‘choosy’ in the selection of their grooming partners; however, such partners are only rarely available at LHW = 0, because only a very brief period after being groomed by *j* ego has a quickly decreasing LIKE_*ij*_ (equal to the decreasing PARTNER˙SAT_*ij*_, see ‘Methods’). This resulted in increasingly lower grooming rates throughout the whole group (compare matrices in top row of Fig. 3B). At increased selectivity (LPS), grooming was still mainly directed up the hierarchy, but was slightly more restricted to similar-ranking partners compared to lower LPS. Note, that due to the very low grooming rates, this last pattern is not visible in Fig. 3B. In the section *‘Grooming Partner Preferences’* further below, this pattern is illustrated clearer.

Next, at intermediate LIKE dynamics (LHW = 180 or 720), LIKE attitudes decreased fast (half-life of 3 HOURS or 1 DAY, respectively) between dyads that do not regularly affiliate, which were mainly the distant-ranked dyads. Increased selectivity (LPS) resulted in increasingly lower grooming rates among distant-ranked dyads (Fig. 3B, matrices in rows 2 and 3). In turn, this reinforced the within-group differences in LIKE attitudes and grooming rates between similar-ranking and distant-ranking dyads (intermediate panels in Figs. 2 and 3B). While similar-ranking individuals became more regular grooming partners, distant-ranking individuals became more incidental grooming partners. At highest selectivity (LPS = 0.99), LIKE attitudes and grooming rates were even differentiated within subsets of similar-ranking dyads. For instance, note that the grooming rates in dyads that differ only 1 in rank, are very different from each other (see the 5th matrix in rows 2 and 3 of Fig. 3B). Still, grooming was directed more up than down the hierarchy. A similar differentiated pattern within similar ranking dyads is visible in the corresponding matrices of LIKE attitudes in Fig. 2. Based on the partner-specific affiliation history individuals differentiated between regular and incidental groomers and this was reinforced by the strong feedback between LIKE attitudes and grooming.

Finally, at slow LIKE dynamics (LHW = 5,400 or 21,600), LIKE attitudes did decrease only very slowly over time and individuals developed high LIKE attitudes towards most other group members, even towards less regular (distant-ranking) groomers (Fig. 2, lower panels). As a result, high selectivity had only a minor effect on grooming rates, as individuals preferred in fact almost any group member as a grooming partner. Therefore, at high LHW, the distribution of grooming rates across different LPS settings changed less compared to intermediate LHW (Fig. 3B, compare rows 2 and 3 with rows 4 and 5). At high LHW, increased LPS resulted in a minor decrease in grooming rates among distant-ranked dyads and grooming was still directed more up the hierarchy (Fig. 3B, especially row 5).

To summarize, in the absence of affiliation partner selectivity (LPS = 0), individuals groomed more up the hierarchy than down the hierarchy and groomed slightly more similar-ranking individuals than distant-ranking ones. High LPS and fast LIKE dynamics (LHW = 0) resulted in very little grooming, due to the lack of partners with high LIKE attitudes. At high LPS and intermediate LIKE dynamics, individuals groomed similar-ranking partners more selectively and grooming became more symmetric. With slow LIKE dynamics (LHW = 21,600), the grooming distribution at high LPS was very similar to low LPS, as individuals developed high LIKE attitudes to most group members.

#### Correspondence of LIKE attitudes and grooming rates

**Figure 4:**
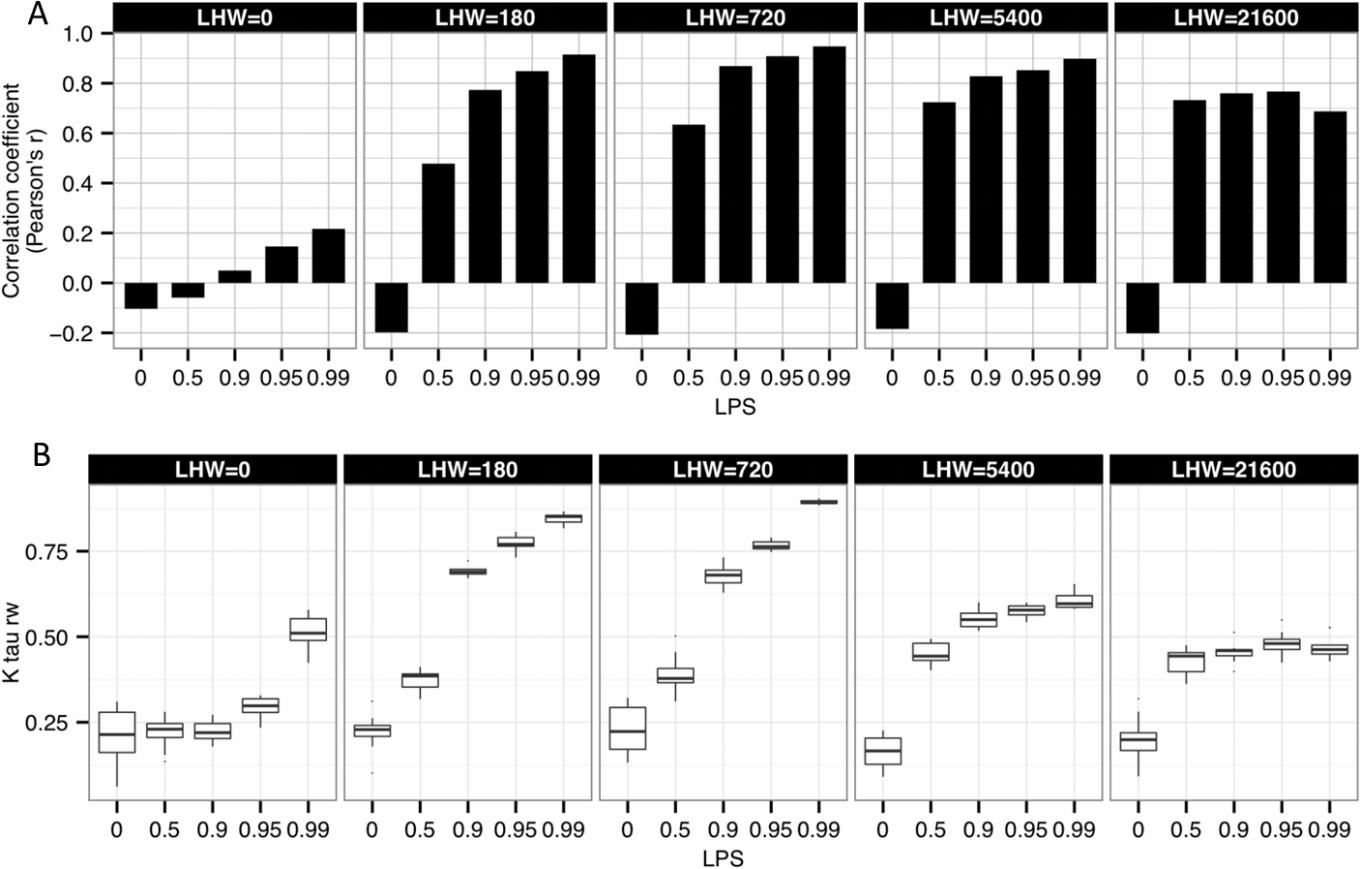
Grooming rates, LIKE attitudes and reciprocity. (A) Correlation between grooming rates and LIKE attitudes. This figure shows the row-wise correlation coefficient (Pearson’s r) between grooming rates and LIKE attitudes for different settings of selectivity (LPS) and LIKE dynamics (LHW). We used matrices of grooming rates and LIKE attitudes averaged over one YEAR. The correlation coefficient was calculated for each of 10 simulation runs and then averaged using a Fisher-z transformation. (B) Reciprocity of the total durations of grooming partner preferences. This figure shows the group-level reciprocity of the total durations of grooming partner preferences for different settings of selectivity (LPS) and LIKE dynamics (LHW). Group-level reciprocity is measured as Kendall rowwise tau. Higher tau_*rw*_ values indicate that stronger reciprocity of preferred partner preference. Taurw values were calculated based on the total partner preference durations averaged over 2 YEARS. The box-plots show the tau_*rw*_ values of 10 simulation runs.

We were interested in how well the LIKE attitudes in our model corresponded to the actual grooming rates of the individuals in our model. LIKE attitudes are an internal, summarized representation of earlier received grooming from other individuals. When comparing visually the matrices of the average LIKE attitudes with the average dyadic grooming rates (Figs. 2 and 3B), it already is clear that for several settings LIKE attitudes did not correspond very well to the dyadic grooming rates. To quantitatively examine the relation between LIKE attitudes and grooming rates, we correlated the LIKE attitudes (averaged over one YEAR) with the dyadic grooming rates (averaged over one YEAR) (Fig. 4A).

As described earlier, at the null model setting (LPS = 0), LHW did not affect the grooming rates, as individuals did not use their LIKE attitudes in this setting. Grooming rates and the decision whom to groom are, in this setting, purely determined by proximity and dominance rank, independently of the LIKE attitudes. Yet, the level of LIKE attitudes did (always) depend on LHW, where higher LHW resulted in higher average LIKE attitudes (see first column in Fig. 2). While grooming was directed up the hierarchy, LIKE attitudes were directed down the hierarchy, as LIKE attitudes were determined by grooming, but did not feed back on grooming. Therefore, at LPS = 0, the correlation between grooming rates and LIKE attitudes was weak and negative, irrespective of LHW (compare the first bars across the panels in Fig. 4A).

Higher partner selectivity (LPS) generally resulted in a higher correlation between LIKE attitudes and grooming rates. This came about due to the stronger feedback between LIKE attitudes and grooming partner choices caused by higher selectivity (LPS). At higher LPS, LIKE attitudes were determined by grooming and did also feedback on future decisions on whom to groom. This resulted in higher reciprocity in grooming rates and LIKE attitudes at higher LPS, compared to lower LPS. As grooming rates and LIKE attitudes were more symmetric at higher LPS, they were also more overlapping and corresponding to each other, resulting in higher correlation coefficients between grooming rates and LIKE attitudes.

Furthermore, intermediate LHW settings resulted in higher correlation coefficients between LIKE attitudes and grooming rates, than low or high LHW, especially when LPS was high (compare the 5 panels in Fig. 4A). At low LHW (LHW = 0), LIKE attitudes decrease too fast to result in a strong feedback from LIKE attitudes to grooming, across all settings of LPS. As no LIKE attitudes could be maintained, LIKE attitudes could also not affect the choice of future grooming partners. Therefore, the correlation between LIKE attitudes and grooming rates was generally very weak at low LHW (Fig. 4A, LHW = 0). At high LHW (LHW = 21,600), LIKE attitudes were almost equally high towards all group members and no strongly preferred partners were available. Although increased selectivity (LPS) causes a stronger feedback between LIKE attitudes and grooming rates, the low variation in LIKE attitudes, which was similar for all LPS between 0.5 and 0.99, resulted in similar correlations between LIKE attitudes and grooming rates (Fig. 4A, LHW = 21,600). Finally, at intermediate LHW, LIKE attitudes corresponded best to grooming rates, given that selectivity was high enough (LPS ≥ 0.9). Here, LIKE attitudes decreased slow enough to incorporate earlier affiliative episodes, but fast enough to enable differentiation between regular and incidental partners. The strong feedback between LIKE attitudes and future grooming partners resulted in highly reciprocal LIKE attitudes and grooming rates for similar-ranking dyads. As a result, the correlation between LIKE attitudes and grooming rates was highest at intermediate LHW (LHW = 720) and high LPS (LPS = 0.99) (Fig. 4A, LHW = 720).

In summary, our model suggests, that (internal) LIKE attitudes correspond most to (external) grooming rates, when individuals are very selective in their partner choice (LPS ≥ 0.9) and when LIKE attitudes incorporated emotional responses to earlier affiliative behavior over intermediate-term periods, i.e., when LHW ranges between 180 and 5400. In these settings we obtained correlation coefficients higher than 0.77 and even up to 0.95 (at LHW = 720 and LPS = 0.99).

### Grooming partner preferences

#### Stability of grooming partner preferences

The main interest of this simulation study is the question whether emotional bookkeeping enables individuals to maintain stable long-term affiliative partner preferences over time. To answer this question, we present the average number of consecutive MONTHS for which an individual remained another individual’s *preferred grooming partner* (i.e., received more than 10% of its total amount of grooming given to others), called the *bout duration of grooming partner preferences*. We also investigated the sum of the durations of all bouts of grooming partner preference per dyad, summed over the total recording period of two YEARS, i.e., the *total duration of grooming partner preferences*.

**Figure 5:**
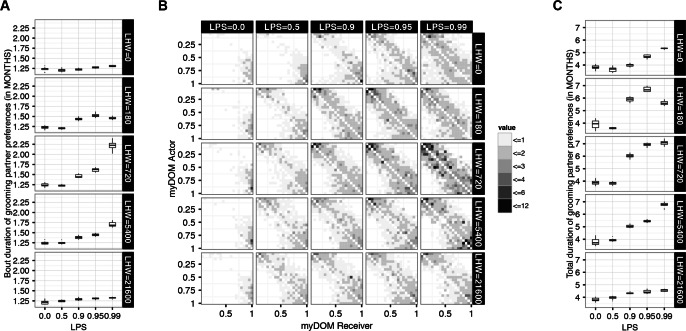
Duration of grooming partnerpreference bouts. (A) This figure shows the group mean of the average preferred partner bout durations for different settings of selectivity (LPS) and LIKE dynamics (LHW). The box-plots show the duration (in MONTHS) of 10 simulation runs, averaged over two YEARS. (B) This figure shows the distribution of the average preferred partner bout durations among the dyads of a group for different settings of selectivity (LPS) and LIKE dynamics (LHW). Partner preferences are directed from actors (*y*-axis) to receivers (*x*-axis), both are ordered by dominance strength, ranging from low-ranking (ID = 1) to high-ranking (ID = 20) individuals. The plot shows the average duration of grooming partner preferences of one example run (the same run as in Fig. 2) in MONTHS, averaged over two YEARS. Darker shades represent longer preferred partner bout durations. Values at the diagonal are by definition not applicable. (C) This figure shows the group mean of the total preferred partner durations for different settings of selectivity (LPS) and LIKE dynamics (LHW). The box-plots show the duration (in months) of 10 simulation runs, averaged over two YEARS.

For most settings of LHW and LPS, the average *bout duration of grooming partner preferences* was between 1.25 and 1.7 MONTHS (Fig. 5A). Only for intermediate LIKE dynamics (LHW = 720) and high selectivity (LPS = 0.99), the average *bout duration of grooming partner preferences* was longer, namely around 2.25 MONTHS (Fig. 5A).

In the null-model setting (LPS = 0), the average *bout duration of grooming partner preferences* was independent of the setting of LHW, as individuals did not use LIKE attitudes to choose grooming partners.

At very fast or very slow LIKE dynamics, increased LPS had only a minor effect on the average *bout durations of grooming partner preferences* (upper and lower panels in Fig. 5A). The lack of group variation in LIKE attitudes in these settings resulted in only a weak feedback from LIKE attitudes to grooming distribution and caused individuals to choose grooming partners mainly based on proximity and/or rank distance.

At intermediate-term LIKE dynamics (LHW) and high selectivity (LPS), individuals developed differentiated LIKE attitudes towards others, based on partner-specific affiliative history. From the average *bout durations of grooming partner preferences* we can conclude that individuals were able to maintain stable grooming partner preferences on average for around 2.25 MONTHS (intermediate panel in Fig. 5A). However, the *bout duration of grooming partner preferences* depended strongly on the rank-distance between the partners; similar-ranking dyads were generally able to maintain longer partner preferences than distant-ranked dyads (intermediate panel of last column in Fig. 5B). At LHW = 720 and LPS = 0.99, for many similar-ranking dyads the average *bout durations of grooming partner preferences* lasted up to 4 MONTHS. Occasionally, *bout durations of grooming partner preferences* in this setting lasted even longer than 12 MONTHS in this setting, with one bout duration in one simulation run lasting even 24 MONTHS.

The total duration of grooming partner preferences was lower than 6 MONTHS for most settings of LPS and LHW (Fig. 5C). This suggests that individuals in these settings changed preferred grooming partners on a regular basis and had no clear predisposition of preferring certain individuals as grooming partners over repeated bouts. At intermediate LIKE dynamics and high selectivity the total duration of grooming partner preferences was generally longer, with the longest total duration of grooming partner preferences of 7 MONTHS at LHW = 720 and LPS = 0.99 (right-most box-plot in third panel of Fig. 5C).

The bout duration and the total duration of partner preferences were longest for LHW = 720 and LPS = 0.99, meaning that in this setting individuals were able to maintain the most stable partner preferences over time. These stable preferences emerge because intermediate-term LIKE dynamics generates a differentiation between regular and incidental groomers and high LPS allows for the selection of regular grooming partners.

#### Reciprocity of grooming partner preferences

We also investigated whether grooming partner preference was reciprocated in our model and how the degree of reciprocity depended on the different settings in our model. To do this, we compared the *total time* that A was a *preferred partner* of B to the *total time* that B was a *preferred partner* of A. We calculated the group level reciprocity of the matrix of *total preferred partner durations*, using Kendall’s tau row-wise matrix correlation. A high tau_*rw*_ indicates a high group-level reciprocity over the total period of 2 YEARS.

When LPS = 0, the reciprocity of grooming partner preferences was lowest (around 0.2), independently of LHW, as here individuals did not use their LIKE attitudes to choose grooming partners (Fig. 4B). At increased selectivity (LPS), reciprocity of grooming partner preferences generally increased for all settings of LHW (Fig. 4B). Thus, at higher selectivity grooming partner preferences became more reciprocated. The feedback from LIKE attitudes to grooming partner choices became stronger at high selectivity. As a result, grooming, LIKE attitudes and also grooming partner preferences became more reciprocated. Reciprocity of grooming partner preferences was strongest (tau_*rw*_ > 0.875) at intermediate LIKE dynamics (LHW = 180 or 720) and highest selectivity (see box-plot at LPS = 0.99 in Fig. 4B, LHW = 180 or 720). At this setting, grooming partner preferences were most stable over time and therefore also most reciprocated.

## Discussion

### Conditions resulting in long-term bonds in the EMO-model

Long-lasting stable reciprocal preferences for particular grooming partners emerged in the EMO-model when two conditions were met: individuals possess an intermediate-term timeframe of emotional bookkeeping, and they are very selective in choosing grooming partners towards whom they assign a high LIKE attitude.

Only a high partner selectivity enabled individuals to differentiate between regular and incidental groomers. However, a strong distinction could only be made when LIKE dynamics were intermediate-term (with a half-life decay in LIKE attitudes of about one ‘day’), but not short-term or long-term, because only this resulted in enough variation in LIKE attitudes between regular and incidental grooming partners. Using this variation as a substrate, high selectivity caused individuals to maintain affiliative relationships with regular groomers and to ‘neglect’ incidental groomers. In turn, differential grooming partner choice reinforced the differentiation of LIKE attitudes. Due to this strong feedback between grooming and LIKE attitudes, both grooming and LIKE attitudes were most reciprocated in this setting and resulted in the longest, most consistent and reciprocal partner preferences.

In contrast, at fast LIKE dynamics (low LHW) the memory of the accumulated emotional response to affiliative events decayed quickly relative to the rate of grooming events. Thus, high LIKE attitudes were maintained only for short periods, which prevented the establishment of a strong feedback between LIKE attitudes and grooming. Therefore, partner preferences were much less reciprocal. At slow LIKE dynamics (high LHW) the memory of accumulated emotional responses to affiliative events decayed slowly; in other words, LIKE attitudes remained high after little grooming and thus towards many individuals, including incidental grooming partners. This resulted in little variation in LIKE attitudes. Consequently, even high partner selectivity did not result in strong partner specificity in interactions. Thus, only intermediate-term LIKE dynamics resulted in low LIKE attitudes towards incidental, and high LIKE attitudes towards regular groomers. Note, that these results depend crucially on the decay rate of the socio-emotional memory (the LIKE attitudes) and the degree of partner selectivity. In a different model study (Campennì & Schino, 2014), individuals are always able to distinguish between better and worse cooperators, even when the difference in their earlier cooperation is very small. In their model, increasing the memory of earlier interactions therefore always increased reciprocity and partner-specificity.

In the absence of any partner selectivity (LPS = 0), affiliation patterns were mainly based on proximity and, to some extent, dominance rank. Selected grooming partners were not consistent in time, as partners changed continuously and arbitrarily. Moreover, partner preferences were not symmetric. At low partner selectivity (low LPS), individuals still interacted with many partners, preventing the maintenance of relationships with preferred partners that were assigned high LIKE attitudes. Only when high partner selectivity limited the number of potential interaction partners, the feedback loop between repeated affiliative events and high LIKE attitudes could emerge and thus also mutually high LIKE attitudes.

If real primates possess LIKE attitudes to integrate the emotional response to earlier received affiliation, it is expected that this internal representation is also reflected in external, observable behavior. In the EMO-model, this was the case in settings that resulted in partner-specific affiliative relationships. Here, the internal representation of an affiliative relationship (LIKE attitude) corresponded best to the externally measurable behavior (grooming). All in all, these model outcomes suggest that settings of intermediate-term LIKE dynamics and high partner selectivity seem most plausible for primates relying on emotional bookkeeping to maintain their social bonds.

### Duration of partner preferences

In the EMO-model, at intermediate-term LIKE dynamics (LHW = 720) the half-life of the partner-specific emotional memory was 720 MINUTES (i.e., 1 DAY). At high partner selectivity (LPS = 0.99) grooming partner preferences were stable across 2.25 MONTHS, on average. In this setting, many similar-ranking dyads maintained their reciprocal grooming partner preferences across 4 MONTHS and occasionally even across one or two YEARS.

Interestingly, the conditions that resulted in long total durations of grooming partner preferences were slightly less specific to the parameter settings: LHW between 180 and 5,400 and LPS between 0.9 and 0.99 yielded comparable total preferred partner durations (Fig. 5C). While at LHW = 720 and LPS = 0.99, this resulted from extended bouts of preferring specific grooming partners, the other settings yielded shorter repeated bouts of maintaining preferred grooming partners. It is yet unclear whether empirical data of primates show a pattern of renewed affiliative bonds due to a predisposition for grooming similar-ranking partners or whether affiliative bonds are continuously upheld over extended periods. Furthermore, the model results indicate that partner similarity, e.g., in dominance rank, may enhance the specificity of emotional bookkeeping. This further suggests that empirical data showing an effect of partner similarity, e.g., concerning dominance rank, does not preclude the involvement of emotional bookkeeping in maintaining partner-specific preferences.

Although the EMO-model yielded partner-specific relationships that lasted for several MONTHS, relationships across one or two YEARS were very rare. Thus, emotional bookkeeping may not be sufficient to generate general long-term partner-specific relationships. Other mechanisms may be required or some specifications of the way emotional bookkeeping was implemented in the model might be modified (see below).

### Does the EMO-model apply to empirical situations?

The conditions under which the EMO-model allows maintaining long-term stable social bonds seem very limiting, since the socio-emotional memory of affiliative events (i.e., the LIKE attitudes) should only last for an intermediate period (half-life of one DAY) and a very high partner selectivity is required.

In the EMO-model, the impact of an affiliative event on the LIKE attitude is independent from the current value of the LIKE attitude (unless this had reached its predetermined maximum value). However, affiliative events may have a stronger effect when the LIKE attitude is low or high, either promoting relationship quality when it is not yet formed or, in contrast, enhancing existing relationships. Recent empirical data suggest the latter possibility, since in wild chimpanzees only grooming with regular partners releases oxytocin (Crockford et al., 2013), suggesting a mechanism of emotional bookkeeping that selectively strengthens existing bonds. In addition, particular affiliative interactions may promote the start of a relationship: in chimpanzees, food sharing increases oxytocin levels in the recipient and the donor of food, even between non-kin with no established bond (Wittig et al., 2014). Thus, particular processes or events may promote the maintenance or start of a social bond. Similarly, particular aversive social events may result in the quick deterioration of a bond, but this effect may again be dependent on the current LIKE attitude. Strong relationships may be more buffered against negative behavior (e.g., more reconciliation among individuals with a good than bad relationship; Aureli & Schaffner, 2002) or against irregular grooming (e.g., Japanese macaques: Schino, di Sorrentino & Tiddi, 2007; ring-tailed coati’s: Romero & Aureli, 2008; capuchin monkeys: Schino & Aureli, 2009; chimpanzees: Gomes, Mundry & Boesch, 2009; long-tailed & rhesus macaques: Massen et al., 2012; but see: long-tailed macaques: Hemelrijk, 1994; Gumert, 2007; chimpanzees: Koyama & Dunbar, 1996; De Waal, 1997; Koyama, Caws & Aureli, 2006; capuchin monkeys: De Waal, 2000). This requires further empirical research.

Also, in the EMO-model there is no limit to the number of individuals assigned a high LIKE attitude. However, empirical data suggest that primates have social bonds with a limited number of group members, called grooming cliques (Kudo & Dunbar, 2001). While often individuals interact with many or all group members (network size is a bit lower or similar to group size), the grooming clique is typically much smaller (Kudo & Dunbar, 2001; Table 1). It has also been observed that after a destabilizing event baboons focus their grooming networks, restricting their grooming partners to the ones they have a close bond with (Wittig et al., 2008). Additional social network analyses in primate species with cohesive groups, e.g., macaques, show that groups often are socially fragmented (Lehmann & Dunbar, 2009). These empirical findings suggest that additional forces, such as partner-specific updates of the social bond and a limitation on the number of partners, may cause differentiation in bonding within the group. These modifications in the updating of LIKE attitudes in the EMO-model may enhance the differentiation between partners, which increases the potential for emotional bookkeeping to maintain partner-specific relationships, also under less extreme partner selectivity conditions.

Finally, the EMO-model makes assumptions regarding the motivational impact of anxiety and satisfaction on the probability to perform affiliative behavior. High anxiety has a relatively stronger effect than low satisfaction on the motivation to execute affiliative behavior. We chose this relative strength to preclude) a direct positive link between satisfaction and the connected LIKE attitude. However, in real animals the relative impact of satisfaction and anxiety may be more similar or even reversed. Primates probably groom not only to reduce anxiety (Dunbar, 2010), since grooming leads to the release of *β*-endorphins and is rewarding for both the receiving and grooming individual (Keverne, Martensz & Tuite, 1989; Dunbar, 2010). The relative importance of anxiety and satisfaction in motivating grooming remains to be established. Altogether, the implementation of the updates of the dynamic LIKE attitudes in the EMO-model is based on conservative assumptions and empirical data suggest that real primates may employ additional processes that enhance partner differentiation.

## Conclusion

In sum, the EMO-model suggests that reciprocal social partner preferences and reciprocal affiliation patterns could result from emotional bookkeeping. In fact, in the EMO-model only emotional bookkeeping generates *partner-specific* affiliation patterns, and they were only found in very specific conditions. Furthermore, in the model, only intermediate-term (not short- or long-term) emotional memory of received affiliation from group members and a strong preference for LIKEd partners led to a strong self-reinforcing feedback between high LIKE attitudes and high grooming rates, which generated reciprocal LIKE attitudes and reciprocal grooming that lasted for prolonged periods of time. The EMO-model employs a relatively conservative manner to update LIKE attitudes and has not incorporated self-reinforcing processes where affiliative interactions with preferred partners exert a stronger LIKE increase than interactions with non-preferred partners. Recent empirical data suggest, however, that such biased updates may actually be present in primates. Since such biased updates will more easily give rise to differentiated relationships with group members, in real primates the conditions that allow emotional bookkeeping to generate prolonged reciprocal affiliative attitudes and behavior may actually be more common than in the EMO-model.

## Supplemental Information

10.7717/peerj.1488/supp-1 Supplemental Information 1 This file contains Figs. F1–F4

10.7717/peerj.1488/supp-2 Supplemental Information 2 This file contains Tables T1–T3

10.7717/peerj.1488/supp-3 Supplemental Information 3 Explanation of behavioral probabilities

## Additional Information and Declarations

### Data Availability

The following information was supplied regarding data availability: The program code of the model is available via the Publications website of the Animal Ecology group (http://www.uu.nl/en/research/animal-ecology/publications).
