# Supplementary material for: Intermediate-term emotional bookkeeping is necessary for long-term reciprocal grooming partner preferences in an agent-based model of macaque groups
Source: PeerJ. 2016 Jan 5;4:e1488. doi: 10.7717/peerj.1488 (PMC4734454; doi:10.7717/peerj.1488)
Supplement: Supplemental Information 1 [file peerj-04-1488-s001.docx]

**Supplementary Material S1**

**
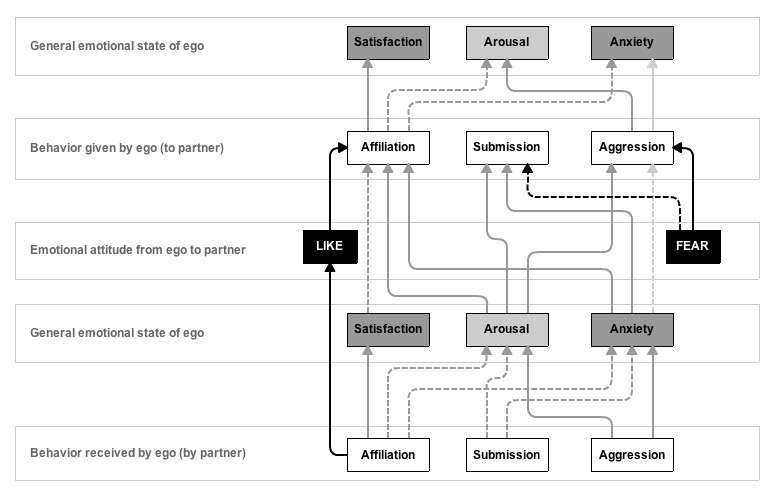
**

**Figure F1: Interactions between behavior, emotional state and attitudes.** This figure illustrates the effect of behavior on an individual’s emotional state and its partner-specific attitudes towards others and vice versa. Solid arrows indicate an increasing effect, while dashed arrows indicate a decreasing effect. Partner-specific effects are depicted as black and general effects are depicted as grey arrows. Light grey arrows depict effects that also depend on other factors, such as the rank of the opponent or the outcome of a fight.

From Evers E et al. (2014) The EMO-Model: An Agent-Based Model of Primate Social Behavior Regulated by Two Emotional Dimensions, Anxiety-FEAR and Satisfaction-LIKE. PLoS ONE 9(2): e87955. doi:10.1371/journal.pone.0087955.g002


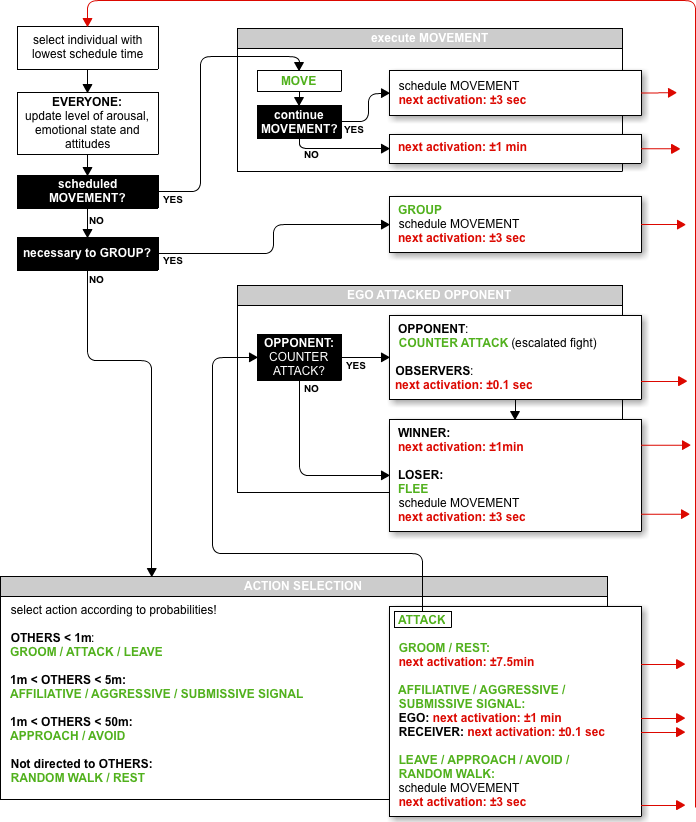


**Figure F2: Process overview of the model.** This figure illustrates the order of the processes executed by the model entities and their timing regime.

From Evers E et al. (2014) The EMO-Model: An Agent-Based Model of Primate Social Behavior Regulated by Two Emotional Dimensions, Anxiety-FEAR and Satisfaction-LIKE. PLoS ONE 9(2): e87955. doi:10.1371/journal.pone.0087955.g001

**
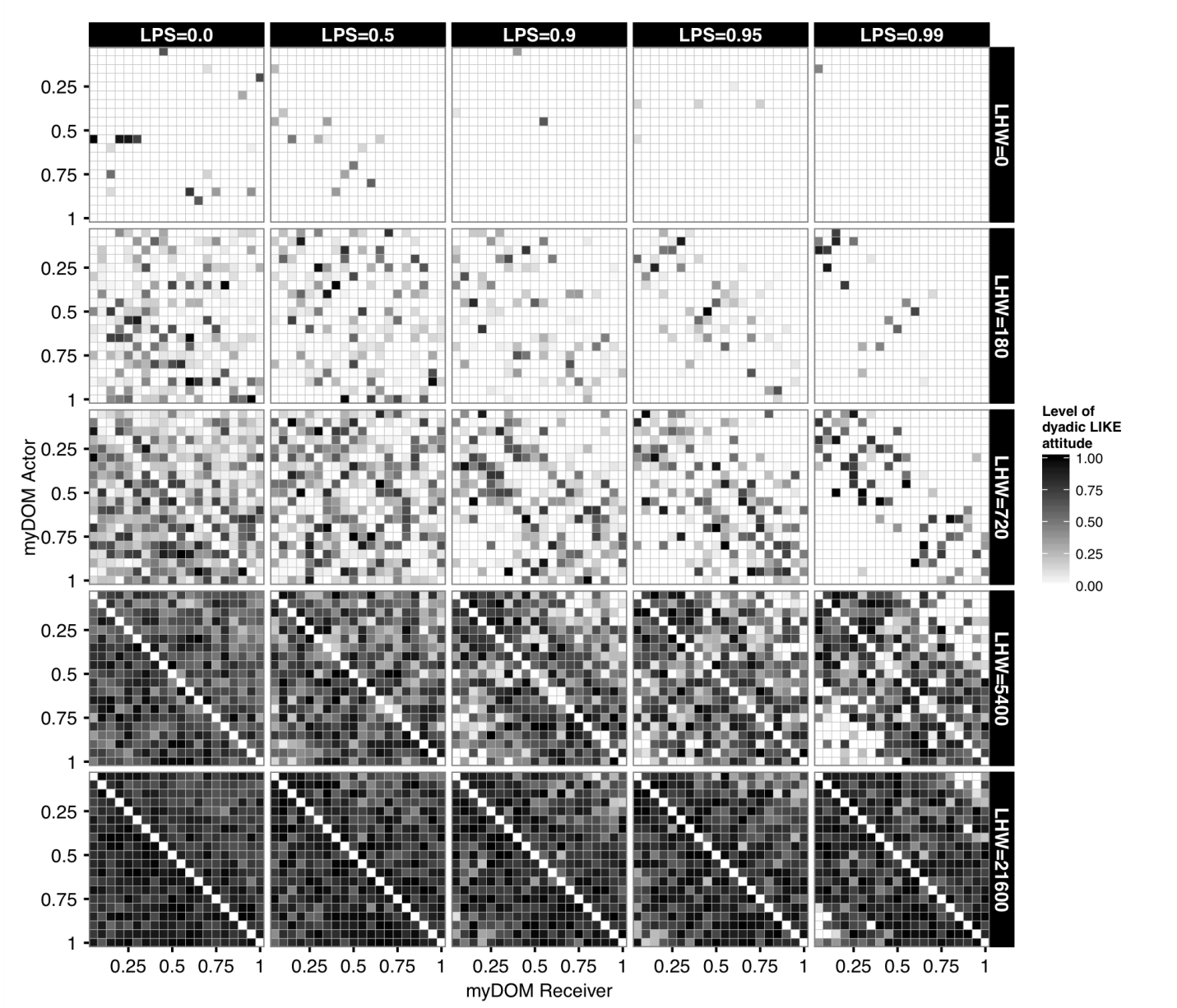
Figure F3**: **Snapshot of LIKE attitudes at TIME=1 YEAR.**

This figure shows the distribution of LIKE attitudes among the individuals of a group for different settings of selectivity (LPS) and LIKE dynamics (LHW). LIKE attitudes are directed from actors (y-axis) to receivers (x-axis), both are ordered by dominance strength, ranging from low-ranking (myDOM=0.05) to high-ranking (myDOM=1.00) individuals. The plot shows the LIKE attitudes of one example run (the same run as in Figure 2) sampled at one point in time (after one YEAR). Darker shades represent higher LIKE attitudes. Values at the diagonal are by definition not applicable.

**
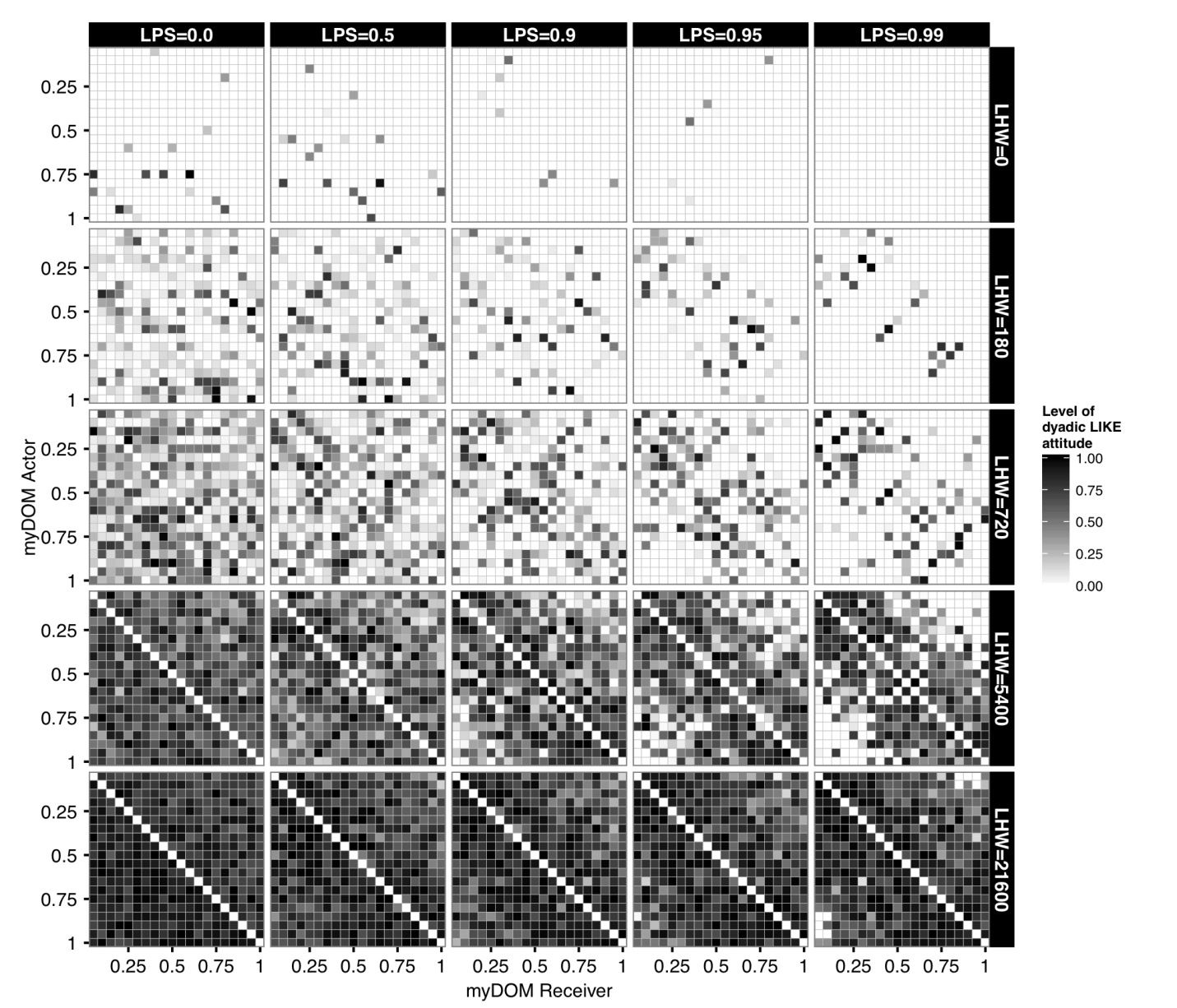
Figure F4**: **Snapshot of LIKE attitudes at TIME=2 YEARS.**

This figure shows the distribution of LIKE attitudes among the individuals of a group for different settings of selectivity (LPS) and LIKE dynamics (LHW). LIKE attitudes are directed from actors (y-axis) to receivers (x-axis), both are ordered by dominance strength, ranging from low-ranking (myDOM=0.05) to high-ranking (myDOM=1.00) individuals. The plot shows the LIKE attitudes of one example run (the same run as in Figure 2 and Figure S2) sampled at one point in time (after two YEARS). Darker shades represent higher LIKE attitudes. Values at the diagonal are by definition not applicable.
