# Supplementary material for: Intermediate-term emotional bookkeeping is necessary for long-term reciprocal grooming partner preferences in an agent-based model of macaque groups
Source: PeerJ. 2016 Jan 5;4:e1488. doi: 10.7717/peerj.1488 (PMC4734454; doi:10.7717/peerj.1488)
Supplement: Supplemental Information 2 [file peerj-04-1488-s002.docx]

**Table T1: Individual-specific state variables of the model entities.**

| **State Variable Name** | **Description** | **(Initial) Value** | **Possible Range or Values** | **Fixed / Dynamic** | **Intended meaning of variable for real life macaques** |
| --- | --- | --- | --- | --- | --- |
| ***GENERAL STATE VARIABLES*** | | | | |  |
| myTIME | Waiting time until next scheduled activation | 1 ± 0.05 min (mean ± SD) | May range between 0.1 ± 0.005 sec (fast reaction) and 7.5 ± 0.375 min (rest/groom) (depending on the social context) | Dynamic | Time between activations |
| myDOM | Dominance strength | between 1/N and 1.0 | Scaled between 1/N (lowest-ranking) and 1.0 (highest-ranking) | Fixed | Dominance rank |
| mySCAN_PROB | Probability of employing scanning | depends on arousal and activity | May range between 0.0 and 1.0 (depending on arousal and activity) | Dynamic | Inclination to look around for presence of other individuals |
| myVIEW_ANGLE | Width of currently employed view angle | 120º | May be either 120º (not scanning) or 360º (scanning) | Dynamic | Looking ahead or looking around |
| ***EMOTIONAL STATE VARIABLES*** | | | | |  |
| myAROUSAL | Arousal state | 0.09 | May range between 0.0 (relaxed) and 1.0 (aroused) | Dynamic | Inclination to act |
| mySATISFACTION | Affiliation-related emotional state | 0 | May range between 0.0 (unsatisfied) and 1.0 (satisfied) | Dynamic | Feeling of satisfaction |
| myANXIETY | Agonism-related emotional state | 0 | May range between 0 (not anxious) and 1 (anxious) | Dynamic | Feeling of anxiety |
| myAROUSAL_LIMIT | Arousal level that is approached over time | 0.09 | May be 0.03 (grooming received), 0.04 (grooming given), 0.09 (default), 0.12 (dominant perceived), 1.0 (maximum) | Dynamic | Inclination to act, depending on social context |
| myANXIETY_LIMIT | Anxiety level that is approached over time | 0 | May be 0.0 (grooming received or given or default) or 1.0 (maximum) | Dynamic | Feeling of anxiety, dep. on social context |
| mySATISFACTION_LIMIT | Satisfaction level that is approached over time | 0 | May be 0.0 (default) or 1.0 (grooming received or given) | Dynamic | Feeling of satisfaction, dep. on social context |
| ***EMOTIONAL ATTITUDE VARIABLES*** | | | | |  |
| FEAR_ij_ | Agonism-related emotional attitude from individual i to j | myDOM_j_ - myDOM_i_ | myDOM_j_ - myDOM_i_ | Fixed | Fear for particular individual |
| LIKE_ij_ | Affiliation-related emotional attitude from individual i to j | 0 | May range between 0 (neutral) and 1 (preferred affiliation partner) | Dynamic | Affiliative feeling for particular individual |

Adapted from Evers E et al. (2014) The EMO-Model: An Agent-Based Model of Primate Social Behavior Regulated by Two Emotional Dimensions, Anxiety-FEAR and Satisfaction-LIKE. PLoS ONE 9(2): e87955. doi:10.1371/journal.pone.0087955.s001.DOC

**Table T2: General and varied model parameters.**

| **Parameter** | **Description** | **Value** |
| --- | --- | --- |
| ***General parameters (fixed)*** | | |
| N | Number of individuals in the group | 20 |
| D | Grid unit | 1 m |
| FIELD_SIZE | Field size | 300 x 300 m |
| MINUTE | Time step | 1 min |
| HOUR | 1 hour | 60 MINUTES |
| DAY | 1 day | 12 HOURS |
| WEEK | 1 week | 7 DAYS |
| MONTH | 1 month | 350/12 DAYS |
| YEAR | 1 year | 50 WEEKS (350 DAYS) |
| ***Varied model parameters*** | | |
| LHW | LIKE-HISTORY WEIGHT, timeframe over which earlier affiliation (LIKE-HISTORY) is incorporated when updating LIKE attitudes | 0, 180, 720, 5400 or 21600 MINUTES |
| LPS | LIKE-PARTNER SELECTIVITY, the degree to which valuable individuals are preferred as affiliation partners | 0.00, 0.50, 0.90, 0.95, 0.99 |
| TIME_STAB_ | Duration of the stabilization period prior to the data-recording period within a simulation | 100, 360, 2700 or 10800 HOURS |
| ***Sensing parameters (fixed)*** | | |
| VIEW_ANGLE | Default view angle | 120º |
| MAX_ANGLE | View angle when scanning | 360º |
| FAR_DIST | Maximum tolerated distance to furthest group member | 100 m |
| MAX_DIST | Maximum distance to individually recognize group members | 50 m |
| NEAR_DIST | Maximum preferred distance to the group | 20 m |
| PERS_DIST | Maximum distance to perceive signals or escalated fights | 5 m |
| INTERACT_DIST | Maximum distance to physically interact with others | 1 m |
| MIN_OTHERS | Minimum preferred number of conspecifics in NEAR_DIST | 3 |
| ***Movement parameters (fixed)*** | | |
| SPEED | Movement speed | 0.6 m/s |
| STOP_CHANCE | Probability of ending the current movement bout | 0.1 |

From Evers E et al. (2014) The EMO-Model: An Agent-Based Model of Primate Social Behavior Regulated by Two Emotional Dimensions, Anxiety-FEAR and Satisfaction-LIKE. PLoS ONE 9(2): e87955. doi:10.1371/journal.pone.0087955.s002.DOC

**Table T3: Effect of social behaviors on arousal, anxiety and satisfaction levels.**

| **Behavior** | **Change of arousal, anxiety or satisfaction level in the model** | **Parameter name** |
| --- | --- | --- |
| ***Behaviors affecting arousal level*** | | |
| Escalated fight observed | + 0.04 | EFO_AR_INC |
| Aggressive signal received | + 0.04 | ASR_AR_INC |
| Attack given | + 0.04 | AG_AR_INC |
| Attack received | + 0.08 | AR_AR_INC |
| Affiliative signal received | - 0.04 | AS_AR_DEC |
| Submissive signal received | - 0.04 | SS_AR_DEC |
| Default decrease | - 0.02 / min | DEF_AR_DEC |
| Default increase | + 0.02 / min | DEF_AR_INC |
| Proximity of dominant | + 0.02 / min | PD_AR_INC |
| Grooming given | - 0.02 / min | GG_AR_DEC |
| Grooming received | - 0.04 / min | GR_AR_DEC |
| ***Behaviors affecting anxiety level*** | | |
| Escalated fight observed | + 0.2 | EFO_ANX_INC |
| Aggressive signal received | + 0.2 | ASR_ANX_INC |
| Attack given | + 0.2 | AG_ANX_INC |
| Attack received | + 0.4 | AR_ANX_INC |
| (Escalated) fight lost | + 0.4 | EFL_ANX_INC |
| Affiliative signal received | - 0.2 | ASR_ANX_DEC |
| Submissive signal received | - 0.2 | SSR_ANX_DEC |
| (Escalated) fight won | - 0.4 | EFW_ANX_DEC |
| Default anxiety decrease rate | - 0.002/min | DEF_ANX_DEC |
| Anxiety decrease rate when giving grooming | - 0.01/min | GG_ANX_DEC |
| Anxiety decrease rate when receiving grooming | - 0.02/min | GR_ANX_DEC |
| ***Behaviors affecting satisfaction level*** | | |
| Satisfaction increase rate when giving grooming | + 0.05/min | GG_SAT_INC |
| Satisfaction increase rate when receiving grooming | + 0.10/min | GR_SAT_INC |
| Default satisfaction decrease rate | - 0.02/min | DEF_SAT_DEC |

In our model, we distinguished between point behaviours, which affect the level of arousal, anxiety or satisfaction instantly (e.g. 'attack received'), and duration behaviours or social contexts, for which the effect on the emotional state depends on the duration the behaviour or context is experienced (e.g. 'grooming received'). From Evers E et al. (2014) The EMO-Model: An Agent-Based Model of Primate Social Behavior Regulated by Two Emotional Dimensions, Anxiety-FEAR and Satisfaction-LIKE. PLoS ONE 9(2): e87955. doi:10.1371/journal.pone.0087955.s005.DOC
